# Preoperative malnutrition is associated with suppressed intratumoral T cell function and distinct tumor-associated microbiota in colorectal cancer: a prospective pilot study

**DOI:** 10.3389/fnut.2026.1802354

**Published:** 2026-05-28

**Authors:** Federica Perillo, Federica Mascaretti, Paola Maragno, Chiara Amoroso, Elena Oriani, Alberto Baeri, Paola Pinco, Luca Pozzi, Ludovica Baldari, Flavio Caprioli, Michele Ghidini, Francesco Strati, Federica Facciotti

**Affiliations:** 1 Department of Biotechnology and Biosciences, University of Milan-Bicocca, Milan, Italy; 2 Gastroenterology and Endoscopy Unit, Fondazione IRCCS Ca’ Granda Ospedale Maggiore Policlinico, Milan, Italy; 3 Department of General and Minimally Invasive Surgery, IRCCS Ca’ Granda, Hospital Maggiore Policlinico, Milan, Italy; 4 Department of Pathophysiology and Transplantation, University of Milan, Milan, Italy; 5 Medical Oncology, IRCCS Ca’ Granda, Ospedale Maggiore Policlinico, Milan, Italy

**Keywords:** CRC, immune system, malnutrition, microbiome, neutrophils

## Abstract

**Introduction:**

Cancer-related malnutrition significantly reduces therapeutic effectiveness, lowers chemotherapy tolerance, and impairs immunotherapy efficacy. Monitoring nutritional status using tools such as the Malnutrition Universal Screening Tool (MUST) may improve clinical outcomes. Malnutrition also profoundly affects immune system functions and gut microbiota composition. However, the relationship between pre-surgery nutritional status, anti-tumor immunity, and tumor-associated microbiota remains poorly understood.

**Methods:**

We prospectively enrolled 43 colorectal cancer (CRC) patients who underwent resection surgery between July 2017 and August 2021 at IRCCS Ca′ Granda Ospedale Maggiore Policlinico in Milan. Patients were evaluated for biochemical, anthropometric, and nutritional profiles, as well as intratumoral immune phenotypes and tumor-associated microbiota. Tumor-associated microbiota analysis was performed in a subset of 8 patients (5 malnourished and 3 non-malnourished) for whom mucosal samples were available.

**Results:**

Malnutrition was associated with increased tissue-infiltrating neutrophils and altered T cell phenotypes, including reduced expression of effector-associated cytokines in conventional T-helper and iNKT cells. Gut microbiota analysis revealed significant associations between neutrophil lymphocyte ratio (NLR) and the bacterial genera *Bacteroides*, *Prevotella*, and *Parabacteroides*, suggesting a potential role for these microbes in shaping immune responses in malnourished individuals.

**Discussion:**

These findings suggest a link between malnutrition, gut microbiota composition, and suppressed anti-tumor immunity in CRC patients.

## Introduction

Malnutrition is a prevalent and critical issue for cancer patients, leading to unintentional weight loss and changes in body composition due to inadequate nutrient intake or absorption (1). This condition severely impacts cancer treatment outcomes by reducing therapy efficacy, increasing toxicity, impairing immunotherapy responses, and deteriorating patients’ quality of life. It also contributes to postoperative complications and, ultimately, threatens survival (2).

The metabolic changes induced by the tumours themselves, or by cancer treatments, can disrupt the body’s ability to effectively utilize nutrients. These complex changes include inflammation, excessive catabolism, futile cycling, and anabolic resistance, all contributing to cachexia, a multifactorial syndrome marked by severe, involuntary loss of skeletal muscle mass, an elevated systemic inflammatory response, and increased protein breakdown (2).

Recent guidelines underlie the importance of early detection and intervention for individuals at nutritional risk. Timely action is crucial to prevent malnutrition’s onset and minimize its devastating impact on clinical outcomes (3). While there is consensus that monitoring nutritional status early can significantly improve clinical trajectories, standardized procedures for assessing and managing malnutrition are not consistently applied across institutions (3). Upon diagnosis, cancer patients should undergo validated nutritional screening with tools such as the Nutrition Risk Screening 2002 (NRS-2002), the Malnutrition Universal Screening Tool (MUST), the Malnutrition Screening Tool (MST), or the Mini Nutritional Assessment Short Form Revised (1, 3, 4). Additionally, combining anthropometric measurements with laboratory assessments, such as C-reactive protein (CRP) (5, 6), albumin (7), and evaluating markers like the neutrophil-lymphocyte ratio (NLR), the accuracy and reliability of nutritional monitoring can be enhanced.

Beyond its direct impact on body composition, malnutrition also negatively affects immune system functions (8), the gut microbiome (9), and overall health. In colorectal cancer (CRC) the immune system functions and the gut microbiome influence its development, progression, and response to therapy (10, 11), but the interconnection of these variables with pre-surgery nutritional status and anti-tumor immunity remains poorly understood.

Here we aim at exploring whether the nutritional status of CRC patients may associate with dysregulated anti-tumor immunity and alterations in the TME. To address this, 43 CRC patients were assessed for their biochemical, anthropometric, and nutritional profiles, alongside intratumoral immune phenotypes and tumor-associated microbiota.

## Materials and methods

### Patients

Patients over the age of 25 with colon cancer disease who underwent resection surgery (*n* = 118) were prospectively enrolled between July 2017 and August 2021 at the Fondazione IRCCS Ca′ Granda Ospedale Maggiore Policlinico of Milan as approved by the Comitato Etico degli IRCCS Istituto Europeo di Oncologia e Centro Cardiologico Monzino, with permission number R 528/16 IEO566 and extended to participating centers (Ethical permission was obtained from the IRCCS associated to the project AIRC granted to FF). Written informed consent was obtained from each participant, all adults, before inclusion. All data were collected at Gastroenterology and Endoscopy Unit of IRCCS Fondazione Ca′ Granda Ospedale Maggiore Policlinico di Milano (Milan, Italy). The study was performed in accordance with the Declaration of Helsinki protocols. Among them, *n* = 43 patients underwent nutrition evaluation and intratumoral immunophenotyping before and after surgery in addition to standard biochemical and anthropomorphic measurement. A flow diagram summarizing patient screening, eligibility, inclusion, and sample availability for immune phenotyping and microbiota profiling is provided in Supplementary Figure 8. These patients were included in the current study and their clinical data are summarized in Table 1.

**Table 1:** Baseline demographic and clinical characteristics.

Variable	Total (*n* = 43)	Non-malnourished patients (*n* = 24)	Moderate or severe malnourished patients (*n* = 19)	*p*-value
Age	67 (59.50; 72.50)	68.50 (63; 77.25)	66 (56.50;71.50)	0.1386
Age at diagnosis	65 (55.50;70.50)	66 (59.50;72.25)	65 (52.50;68.50)	0.1705
≥ 50	38 (88.4%)	23 (95.8%)	15 (78.9%)	0.1529
< 50	5 (11.6%)	1 (4.2%)	4 (21.1%)	
Sex				
Female	22 (51.2%)	11 (45.8%)	11 (57.9%)	0.6322
Male	21 (48.8%)	13 (54.2%)	8 (42.1%)	
BMI	23.8 (20.93;25.8)	24.82 (22.79;27.13) Min 20.76 Max 45.92	20.76 (19.94;23.84) Min 18.43 Max 33.20	0.0009
Weight (Kg)	61 (57;76)	69 (59;79) Min 55 Max 130	59 (55;67) Min 45 Max 85	0.02263
Tumor localization				
Right	14 (32.6%)	8 (33.3%)	6 (31.6%)	0.7673
Left	11 (25.6%)	7 (29.2%)	4 (21.1%)	
Rectum	18 (41.8%)	9 (37.5%)	9 (47.3%)	

This table for continuous variables, the median, first and third quartiles are reported; for categorical variables, the percentage with respect to the total per group. To test the difference between non-malnourished patients (MUST = 0) and Moderate or severe malnourished patients (MUST = 1/2/3), the nominal *p*-value by Mann–Whitney was computed for continuous variables, the nominal *p*-value by Pearson’s Chi-squared test with Yates’ continuity correction or by Fisher’s exact test was computed for categorical variables.

### Anthropometric measurements

Anthropometric measurements, obtained from medical records, consisted of weight, height and the Body Mass Index (BMI). These data are summarized in Table 1 and Supplementary Table 1.

The BMI for adults was classified according to criteria established by the World Health Organization. Cut-off points of <18.5 kg/m^2^ for malnourished, 18.5 kg/m^2^–24.9 kg/m^2^ for normal weight, >25 kg/m^2^ for overweight, and >30 kg/m^2^ for obesity were considered for statistical analysis (12).

### MUST evaluation

Patients were evaluated for the Malnutrition Screening Tools (MUST) and categorized in: 0 (low risk of malnutrition), 1 (moderate risk of malnutrition), ≥ 2 (high risk of malnutrition) (7). Percentage weight loss was calculated from the patient’s weight at the date of surgery compared to the patient’s usual weight.

### Biochemical measurements

Biochemical measurements were performed from venous blood samples at the time of the surgery and two months after, including albumin, C-reactive protein (CRP), total proteins, neutrophils and lymphocyte counts. These last two values were used to calculate the neutrophil–lymphocyte ratio (NLR) used to predict prognosis (6). Patient’s biochemical and immunological data are summarized in Table 2 and Supplementary Table 2.

**Table 2:** Biochemical and immunological features.

Variable	Total (*n* = 43)	Non-malnourished patients (*n* = 24)	Moderate or severe malnourished patients (*n* = 19)	*p*-value
Albumin g/dL (operation)	4.34 (3.95; 4.53)	4.2 (4.1; 4.56)	4.37 (3.75; 4.5)	0.766
Total protein g/dL (operation)	6.9 (6.6; 7.15)	6.9 (6.6; 7.01)	6.75 (6.33; 7.18)	0.4966
Total protein g/dL (after 2 months)	7.35 (6.8; 7.58)	7.55 (6.98; 7.68)	7.05 (6.75; 7.33)	0.285
Neutrophils 10e9/L (operation)	6.87 (4.89; 8.73)	6.61 (4.85; 8.5)	7.38 (5.27; 8.73)	0.6749
Neutrophils 10e9/L (after 2 months)	3.15 (2.33; 4.26)	3.22 (2.36; 5.43)	2.61 (2.17; 4.09)	0.2482
Lymphocyte 10e9/L (operation)	1.12 (0.95; 1.56)	1.26 (0.98; 1.61)	1 (0.93; 1.41)	0.2799
Lymphocyte 10e9/L (after 2 months)	1.97 (1.54; 2.3)	2.02 (1.6; 2.69)	1.69 (1.34; 2.2)	0.2359
CRP mg/dL (operation)	8.81 (5.94; 14.76)	9.05 (6.32; 14.81)	6.67 (4.03; 11.49)	0.5972
CRP mg/dL (after 2 months)	0.41 (0.13; 1.19)	0.58 (0.11; 1.19)	0.31 (0.17; 1.49)	0.9591
Neutrophils/Lymphocyte (operation)	6.41 (4.03; 8.73)	6.09 (3.75; 8)	6.94 (4.66; 10.29)	0.2834
Neutrophils/Lymphocyte (after 2 months)	1.58 (1.23; 2.72)	1.74 (1.16; 2.79)	1.45 (1.27; 2.02)	0.6415
Vascular invasion				
Yes	16 (30.77%)	8 (33.33%)	8 (42.11%)	0.7846
No	27 (69.23%)	16 (66.67%)	11 (57.89%)	
Lymphovascular invasion				
Yes	1 (2.33%)	0	1 (5.26%)	0.4419
No	42 (97.67)	24 (100%)	18 (95.74%)	
Perineural invasion				
Yes	13 (30.23%)	6 (25%)	7 (36.84%)	0.6133
No	30 (69.77%)	18 (75%)	12 (63.16%)	
Lymphocytic infiltration				
Yes	29 (67.44%)	18 (75%)	11 (57.89%)	0.3892
No	14 (32.56%)	6 (25%)	8 (42.11%)	

This table for continuous variables, the median, first and third quartiles are reported; for categorical variables, the percentage with respect to the total per group. To test the difference between Non-malnourished patients (MUST = 0) and Moderate or severe malnourished patients (MUST = 1/2/3), the nominal *p*-value by Mann–Whitney was computed for continuous variables, the nominal *p*-value by Pearson’s Chi-squared test with Yates’ continuity correction or by Fisher’s exact test was computed for categorical variables.

### Prognostic index calculation

The Prognostic Nutritional Index (PNI) was calculated with the following formula: albumin level (g/L) + 0.005 × total lymphocyte count/mm^3^ (7). Cut-off point <45 was suggested to predict risk of surgical complications (13).

### Predictive modeling of malnutrition risk

A Logistic Regression classifier was trained to predict the level of malnutrition based on five different predictors: age, neutrophil levels (10^9/L) at surgery, neutrophil levels (10^9/L) two months post-surgery, lymphocyte levels (10^9/L) two months post-surgery and NLR at surgery. These variables were selected using Lasso regression run with 10-folds cross validation. Specifically, a unitary increase in each of these variables raises the malnutrition risk by 0.94, 1.38, 0.50, 0.25 and 0.99 times, respectively. The Logistic Regression model trained using these five variables had a sensitivity = 0.74, specificity = 1, misclassification rate = 0.09, accuracy = 0.91, precision = 1, *p* = 0.01, F1 score = 0.85, AIC = 56.6, BIC = 67.27, pseudo-R^2^ (McFadden) = 0.24, pseudo-R^2^ (Cragg-Uhler) = 0.38, *p* = 0.01, χ^2^(5) = 14.33.

### LPMCs isolation

Lamina propria mononuclear cells (LPMCs) were isolated as previously described (14). Briefly, tumour samples were taken transversally to collect both marginal and core tumour zone. Normal adjacent tissues were sampled at least 10 cm from the tumour margin. The dissected intestinal mucosa was freed of mucus and epithelial cells in sequential steps with Dithiothreitol (DTT) (0.1 mmoL/L) and EDTA (1 mmoL/L) (Sigma-Aldrich) and then digested with collagenase D (400 U/mL) (Worthington Biochemical Corporation) for 5 h at 37 °C in agitation. Lamina propria mononuclear cells (LPMCs) were then separated with a Percoll gradient.

### Cytofluorimetric analysis

Immune cells were washed and stained with the combination of monoclonal antibodies purchased from different vendors, as listed in Supplementary Table 3. LPMCs were stained and iNKT cells were identified using human CD1d: PBS57 Tetramer (NIH Tetramer core facility) diluted in PBS with 1% heat-inactivated FBS for 30 min at 4 °C. For intracellular cytokine labelling, cells were incubated for 3 h at 37 °C in RPMI-1640 + 10% FBS with PMA (50 ng/mL, Merck), Ionomycin (1 μg/mL, Merck) and Brefeldin A (10 μg/mL, Merck). Cells were fixed and permeabilized using Cytofix/Cytoperm (BD) before the addition of the antibodies detecting the cytokines produced upon stimulation. Samples were analysed with a FACSLyrics flow cytometer (BD Biosciences). Data were analysed using the FlowJo software (Version 10.8, BD) gated to exclude singlets based on light scatter.

### 16S rRNA gene sequencing and data analysis

The microbiota associated with tumor lesions (TUMs) and adjacent non-tumor colon tissue (NCT) was collected during surgery by scraping the intestinal mucus from tissue samples, collected in TES buffer (50 mM Tris–HCl pH 7.5, 10 mM NaCl, 10 mM EDTA) and stored at −80 °C. The bacterial DNA was extracted with GENOME DNA isolation kit (MP Biomedicals) following the manufacturer’s instructions. 16S rRNA gene amplification, purification, library preparation, and paired-end sequencing using the Illumina MiSeq platform were performed as previously described (15). We used the ZymoBIOMICS Microbial Community Standard II (mock community) and negative controls to quantify and mitigate biases introduced during 16S rRNA gene sequencing procedures. Reads were pre-processed using the MICCA pipeline (v.1.7.2) (16, 17). Forward and reverse reads were merged setting a minimum overlap length of 32 nucleotides and a maximum number of allowed mismatches equal to 8. Primers were trimmed using micca trim and reads with length higher than 400 base pairs or error rate higher than 1% were removed via micca filter. Filtered sequences were denoised using the UNOISE algorithm implemented in micca, removing all chimera sequences, to determine true biological sequences at the single nucleotide resolution by generating ASVs. Bacterial ASVs were taxonomically classified using micca classify and the Ribosomal Database Project (RDP) Classifier v2.12, a naïve Bayesian classifier (18). Multiple sequence alignment of 16S sequences was performed using the Nearest Alignment Space Termination (NAST) algorithm (19) implemented by micca msa with the template alignment clustered at 97% similarity of the Greengenes core set (20). Phylogenetic trees were inferred using micca tree. The number of reads per sample varied between 16,722 and 158,549; sampling heterogeneity was reduced rarefying samples, using micca tablerare, at a rarefaction threshold of 16,722 reads per sample, equivalent to the depth of the less abundant sample. Eventually, micca tabletotax was used to summarize communities by their taxonomic composition. All the subsequent analyses were performed in R environment using phyloseq R package (v1.48.0) (21). The mean relative abundances across samples of these genera were computed separately for Non-malnourished and Moderate or severe malnourished patients, both for healthy and tumor tissues, and plotted using ggplot2 R package (v3.5.1) (22). Alpha (within-sample richness evaluated computing the Observed richness and the Shannon index) and beta-diversity (between-sample dissimilarity evaluated computing the Bray-Curtis distance) estimates were computed using phyloseq R package. Alpha diversity values were tested using wilcox_test function of rstatix R package (v0.7.2) (23) considering separately paired and unpaired samples. Permutational multivariate analysis of variance (PERMANOVA) test was performed using adonis function in the vegan R package (v2.6.6.1) (24, 25) with 999 permutations to test beta diversity values. The differential abundance of the different ASVs – comparing Non-malnourished and Moderate or severe malnourished patients, considering separately healthy and tumor samples – were tested using DESeq2 R package (v1.44.0) (25) on the non-rarefied data. *p*-values were false discovery rate corrected using the Benjamini–Hochberg procedure implemented in DESeq2. The results were represented in a volcano plot generated by ggplot2 R package, only the ASVs with a nominal *p*-value < 0.05 were colored, and the name was reported only for the subset with a nominal *p*-value < 0.05 and an absolute log2FC > 1. The Spearman’s correlation test between the abundances of the ASVs differently enriched between the two groups of patients – separately in healthy and tumor samples—and the abundances of the different biochemical molecules and LMPCs was computed using corr.test function from psych R package (v2.5.3). The resulting correlation values were represented in a heatmap using pheatmap R package (v1.0.12) (26).

### Statistical analysis

Patients were divided in two groups based on the level of malnutrition: non-malnourished (MUST = 0) (*n* = 24) and moderate or severe malnourished patients (MUST ≥ 1) (*n* = 19). The distributions of clinical data in the different groups were represented as bar plots and violin plots; Chi-squared and Mann Whitney tests were used to evaluate significant differences. The mean abundance of each biochemical molecule in the patients of each group was computed and represented in R environment (v4.4.1). In addition, the Spearman’s correlation between the abundances of each biochemical molecule and the MUST variable was computed using corr.test function from psych R package (v2.5.3) and represented separately for the two time points: the time of the surgery and two months after. The mean relative abundance and standard error of each immune cell type were computed and significant differences in abundances were evaluated with Mann Whitney test (nominal *p*-values < 0.05 were reported).

To build a logistic regression model to predict the probability of a patient being malnourished, only some of the variables—those considered to have more clinical impact—were evaluated: sex, age, albumin levels (g/dL) at surgery, total protein levels (g/dL) at surgery, neutrophil levels (10^9/L) at surgery, neutrophil levels (10^9/L) two months post-surgery, lymphocyte levels (10^9/L) at surgery, lymphocyte levels (10^9/L) two months post-surgery, location of the disease, stage of the disease, use of adjuvant chemotherapy, PCR levels (mg/dL) at surgery, PNI, NLR at surgery and NLR two months post-surgery. The number of missing values was computed for each variable, and it varied between 0 and 6, among the 43 observations. Being a limited number, we decided to replace missing values with the average value of the variable in patients with the same level of malnutrition. Two different approaches of feature selection were used, best subset selection using complete enumeration [via bestglm method from bestglm R package (v0.37.3)] (27) and Lasso regression with cross validation [using cv.glmnet method from glmnet R package (v4.1.9)] (28). These methods were applied on the whole dataset, without dividing in train and test set, due to the limited sample size. Best subset selection method was run using AIC as information criterion to find the best fit. The best model suggested by this method included eleven variables: age, albumin levels (g/dL) at surgery, neutrophil levels (10^9/L) at surgery, neutrophil levels (10^9/L) two months post-surgery, lymphocyte levels (10^9/L) at surgery, lymphocyte levels (10^9/L) two months post-surgery, stage of the disease, use of adjuvant chemotherapy, PCR levels (mg/dL) at surgery, PNI and NLR at surgery. However, the logistic regression model trained using glm method from stats R package (v4.4.1) (29) using these variables showed a clear overfitting problem, having sensibility, specificity and AUC all equal to 1. Lasso regression run with 10-folds cross validation identified a minimum lambda value of 0.065 that minimized the mean squared error. When lambda is equal to the logarithm of this minimum lambda value, only five variables have a coefficient different from zero: age, neutrophil levels (10^9/L) at surgery, neutrophil levels (10^9/L) two months post-surgery, lymphocyte levels (10^9/L) two months post-surgery and NLR at surgery. A logistic regression model was trained using glm method from stats R package (v4.4.1) (29) using these five variables. The logistic regression assumptions were tested for this model: first, Box-Tidwell test was computed to test linearity using boxTidwell function from car R package (v3.1.3) (30). All the five selected variables had a *p*-value from Box-Tidwell test higher than 0.3, indicating that the relationship between the predictor and the logit could be considered linear. Then, the presence of influential values was evaluated computing the Cook’s distance by augment function of broom R package (v1.0.8) (31); despite some points had a high Cook’s distance, none of them was considered outlier since their absolute standardized residuals were always below 3. Eventually, multicollinearity was tested using vif function from car R package (v3.1.3) (30): since all variables had a value of VIF below 3, collinearity was excluded.

The same analyses were performed by dividing patients into three malnutrition groups: non-malnourished (MUST = 0) (n = 24), moderately malnourished (MUST = 1) (n = 10), and severely malnourished patients (MUST ≥ 2) (n = 9). Chi-squared and Kruskal-Wallis tests were used to evaluate significant differences.

## Results

### Demographic and clinical variables do not predict malnutrition in CRC patients

**Figure 1:**
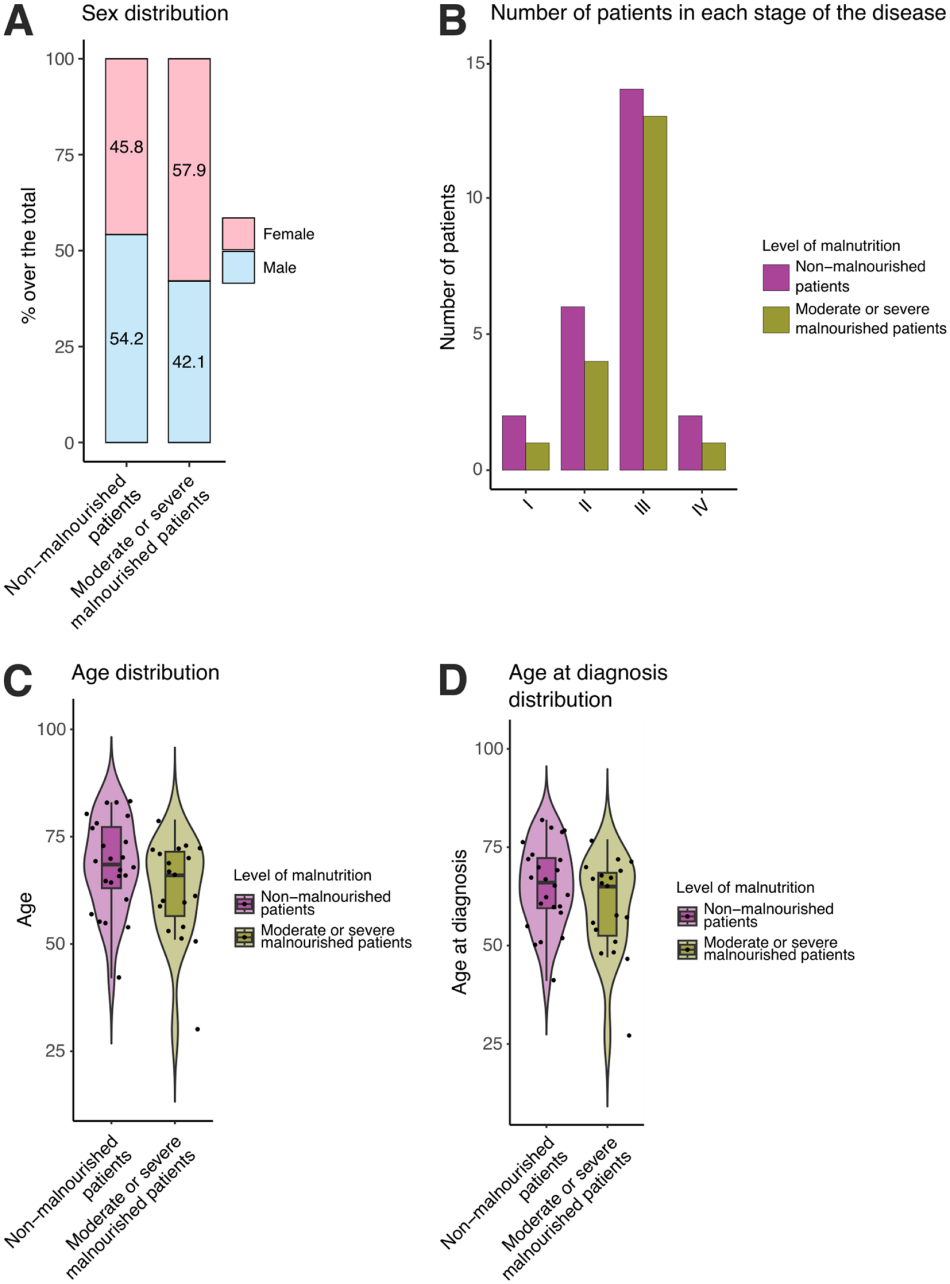
**(A)** Bar plot representing the relative distribution of male (blue) and female (pink) in non-malnourished patients (*n* = 24) and in moderate or severe malnourished patients (*n* = 19). **(B)** Bar plot representing the numbers of non-malnourished (violet) and moderate/severe malnourished patients (green) in the four stages of the disease (I *n* = 3, II *n* = 10, III *n* = 17, IV *n* = 3). **(C)** Violin plot representing the age distribution in non-malnourished patients (*n* = 24; violet) and in moderate or severe malnourished patients (*n* = 19; green). **(D)** Violin plot representing the age at diagnosis distribution in non-malnourished patients (*n* = 24; violet) and in moderate or severe malnourished patients (*n* = 19; green).

Several studies have highlighted a notable survival advantage for female CRC patients under 65 years of age, which may be partly attributed to the protective effects of sex hormones (32–36). Conversely, factors like advanced age and malnutrition are associated with increased perioperative mortality, a higher rate of complications, and overall poorer prognosis (37). Patients were assessed using the Malnutrition Screening Tools (MUST) and categorized as non-malnourished (score = 0), or moderate/severe malnourished (score ≥ 1) (Tables 1, 2). Then, we determined whether a correlation may exist between nutrition status and age, sex, and disease stage. We observed no relationships between demographic factors and malnutrition. In fact, we observed that both male and female patients were well represented across the two nutritional groups (Figure 1A), with the majority of participants being affected by stage III cancer, regardless of their nutritional status (Figure 1B). Moreover, no significant differences were observed between age, age at diagnosis, and nutritional status (Figures 1C,D). The same cohort was also stratified into three groups based on their MUST score: non-malnourished (score = 0), moderately malnourished (score = 1), and severely malnourished (score ≥ 2). No significant differences were found in terms of sex, age, or age at diagnosis, with the majority of patients across all groups being in stage III of the disease (Supplementary Figures 1A–D; Supplementary Table 1).

Malnutrition is a complex condition, as for example also overweight or obese cancer patients can be malnourished (4). Anthropometric measurements revealed a correlation between nutritional status, BMI, and weight distribution. Both BMI and weight tended to decrease in patients at moderate or severe risk of malnutrition (Supplementary Figures 2A–D).

Together, these findings suggest that, while certain demographic and clinical characteristics can individually influence CRC patient disease outcomes, they do not appear to be directly correlated with nutritional status. On the other hand, anthropometric measurements emerge as critical indicators in assessing malnutrition risk in CRC patients.

### Inflammatory biomarkers show trends of association with nutritional status in CRC patients

**Figure 2:**
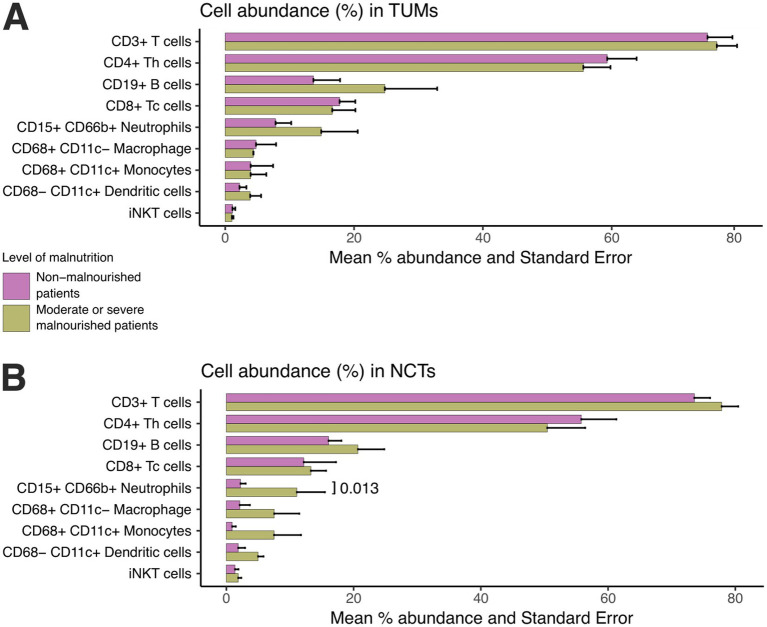
**(A)** Spearman’s correlation between malnutrition risk, expressed as the MUST score, and different biochemical markers of inflammation measured at the time of surgical intervention. Bars are colored based on the Spearman’s correlation value. There is not any statistically significant correlation. **(B)** Spearman’s correlation between malnutrition risk, expressed as the MUST score, and different biochemical markers of inflammation measured two months after surgical intervention. Bars are colored based on the Spearman’s correlation value. There is not any statistically significant correlation. **(C)** Comprehensive representation of the mean values of different biochemical markers of inflammation, measured at the time of surgical intervention and two months after, in patients grouped by level of malnutrition (not malnourished, violet, moderate or severe malnourished green).

Numerous studies have highlighted the role of serum inflammatory markers in predicting cancer prognosis (38). To explore if these markers are linked to patients’ nutritional status, we examined their relationship with malnutrition risk, as determined by the MUST score. No significant correlation was found between the MUST score and traditional nutritional markers like albumin (38), either at surgery or two months post-surgery (Figures 2A,B). Similarly, lymphocyte counts showed no correlation with MUST score, as well as other biochemical markers evaluated (Figures 2A,B).

The cancer-associated systemic inflammatory response is often marked by an increase in circulating neutrophil counts, which promotes tumor progression through the secretion of cytokines and chemokines (38). In contrast, lymphocytes play a key role in mounting a cytotoxic immune response against cancer, and a decrease in lymphocyte counts has been shown to negatively impact CRC prognosis (38). We found a weak (r = 0.15), but not significant positive tendency between the MUST score and the NLR at surgery (Figure 2A). This suggests that patients with higher malnutrition risk may also have a more severe inflammatory condition at the time of surgery.

To further investigate this issue, we compared the mean values of these inflammatory markers between non-malnourished and moderate/severe malnourished patients, finding no significant differences (Figure 2C). However, when patients were categorized into three groups based on their MUST score (score = 0, 1, ≥ 2), we observed an increase, even if not significant, in CRP levels in moderately malnourished patients as compared to the other groups (Supplementary Figure 3; Supplementary Table 2) suggestive of the presence of a more severe inflammatory condition.

To evaluate the predictive power of inflammatory markers on nutritional status, we developed a Logistic Regression classifier. Using Lasso regression, we identified age, NLR, and perioperative neutrophil and lymphocyte levels as the most significant predictors. The model demonstrated high diagnostic accuracy (91%) and strong specificity (1.00), effectively distinguishing malnourished from non-malnourished patients (AUC = 0.836, Supplementary Figures 4A,B).

Overall, these findings suggest a link between the nutritional status of CRC patients undergoing resection surgery and their inflammatory condition, which can be assessed through serum molecular and cellular markers. This insight could provide a better understanding of how malnutrition impacts cancer prognosis and treatment outcomes.

### Malnourished CRC patients exhibit increased immune infiltration and pro-tumor phenotypes in CD4+ and iNKT cells

The immune landscape within the tumor microenvironment (TME) plays a critical role in shaping cancer progression and prognosis. Immune cells in the TME, both myeloid and lymphoid, exhibit complex dual functions, either suppressing tumor growth through pro-inflammatory cytokine production and direct tumor cell killing or promoting tumor progression through angiogenesis and invasion by supporting cell proliferation (39). To better understand how the TME is affected by patients’ nutrition status, we immunophenotyped by multidimensional single-cell flow cytometry both tumor lesions (TUMs) and adjacent non-tumor colon tissue (NCT) of malnourished and non-malnourished patients.

**Figure 3:**
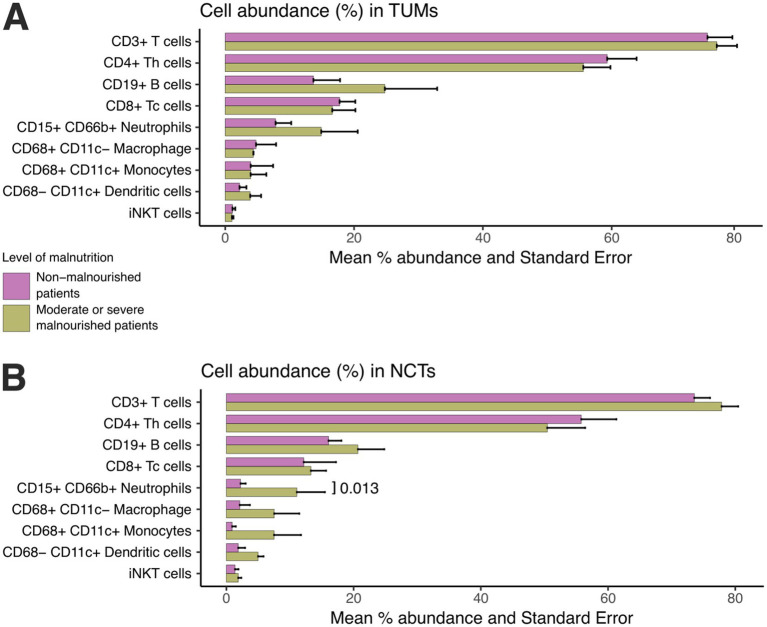
**(A)** Bar plot representing mean frequency and standard error of different immune cellular population analyzed by FACS infiltrating the tumor samples (TUM) of non-malnourished (violet) and moderate or severe malnourished patients (green). **(B)** Bar plot representing mean frequency and standard error of different immune cellular population analyzed by FACS infiltrating the adjacent non-tumor colon tissue (NCT) of non-malnourished (violet) and moderate or severe malnourished patients (green). Nominal *p*-values from Mann Whitney test are reported only if significant (≤ 0.05).

We observed that TUM lesions in patients at higher risk of malnutrition did not show marked differences in overall immune cell infiltration when compared to non-malnourished patients (Figure 3A). However, in the NCT context, malnourished patients exhibited a significant increase in CD15^+^ CD66b^+^ neutrophils (Figure 3B). These results were further validated by categorizing patients into three groups based on the MUST score. While immune infiltrate levels in TUMs showed minimal variation between groups (Supplementary Figure 5A), moderately malnourished patients (MUST score = 1) displayed a significantly higher presence of neutrophils in NCT tissues (Supplementary Figure 5B). These findings suggest that neutrophils are more likely to infiltrate the tissues of malnourished patients, particularly in distal regions, potentially affecting both the tumor core and margins.

**Figure 4:**
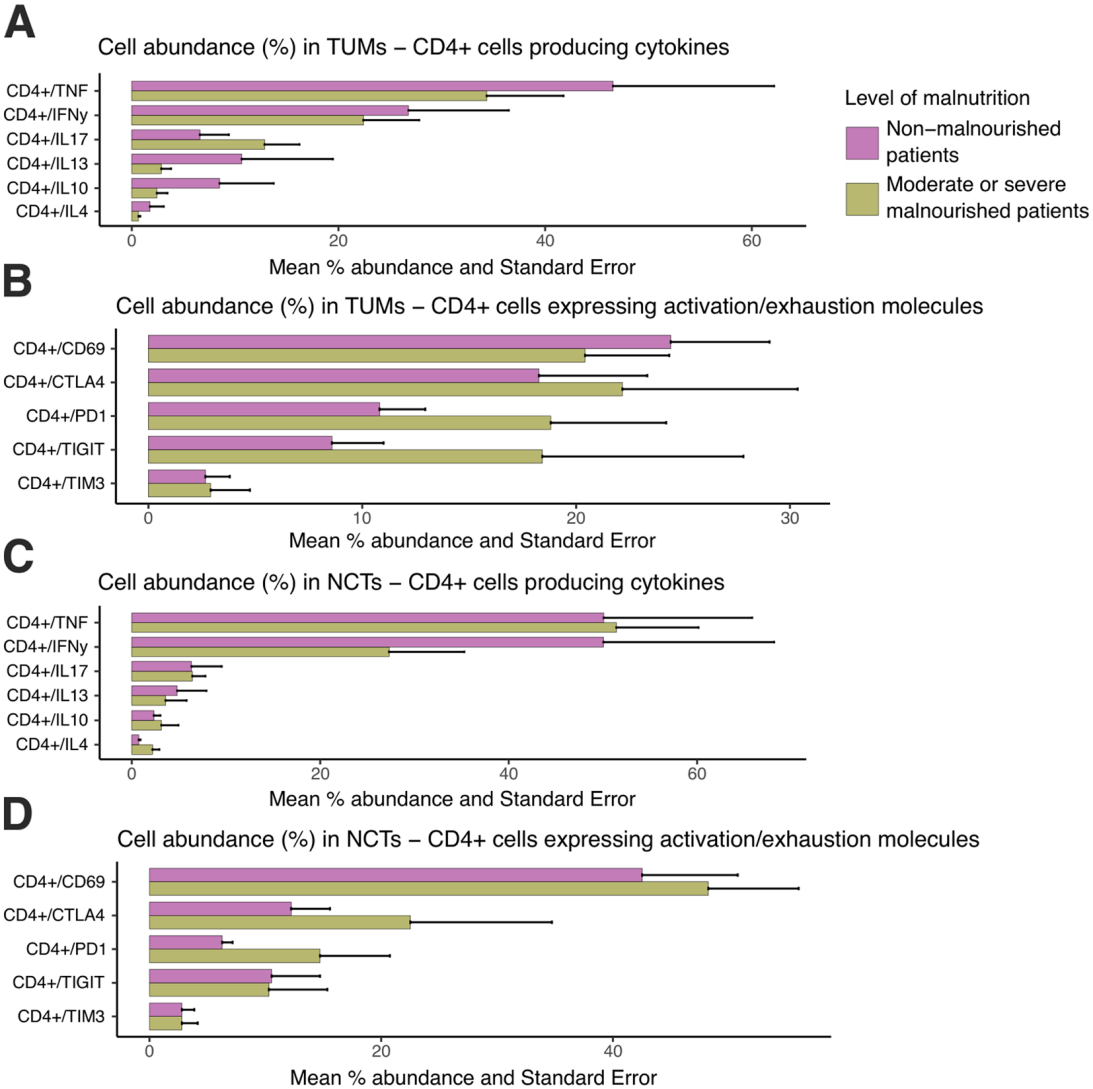
**(A)** Bar plot representing mean frequency and standard error of CD4^+^ cells producing different cytokines in tumor samples (TUM) of non-malnourished (violet) and moderate or severe malnourished patients (green). **(B)** Bar plot representing mean frequency and standard error of CD4^+^ cells expressing different activation/exhaustion molecules in tumor samples (TUM) of non-malnourished (violet) and moderate or severe malnourished patients (green). **(C)** Bar plot representing mean frequency and standard error of CD4^+^ cells producing different cytokines in adjacent non-tumor colon tissue (NCT) of non-malnourished (violet) and moderate or severe malnourished patients (green). **(D)** Bar plot representing mean frequency and standard error of CD4^+^ cells expressing different activation/exhaustion molecules in adjacent non-tumor colon tissue (NCT) of non-malnourished (violet) and moderate or severe malnourished patients (green). Differences were evaluated with Mann Whitney test; no significant differences were detected.

**Figure 5:**
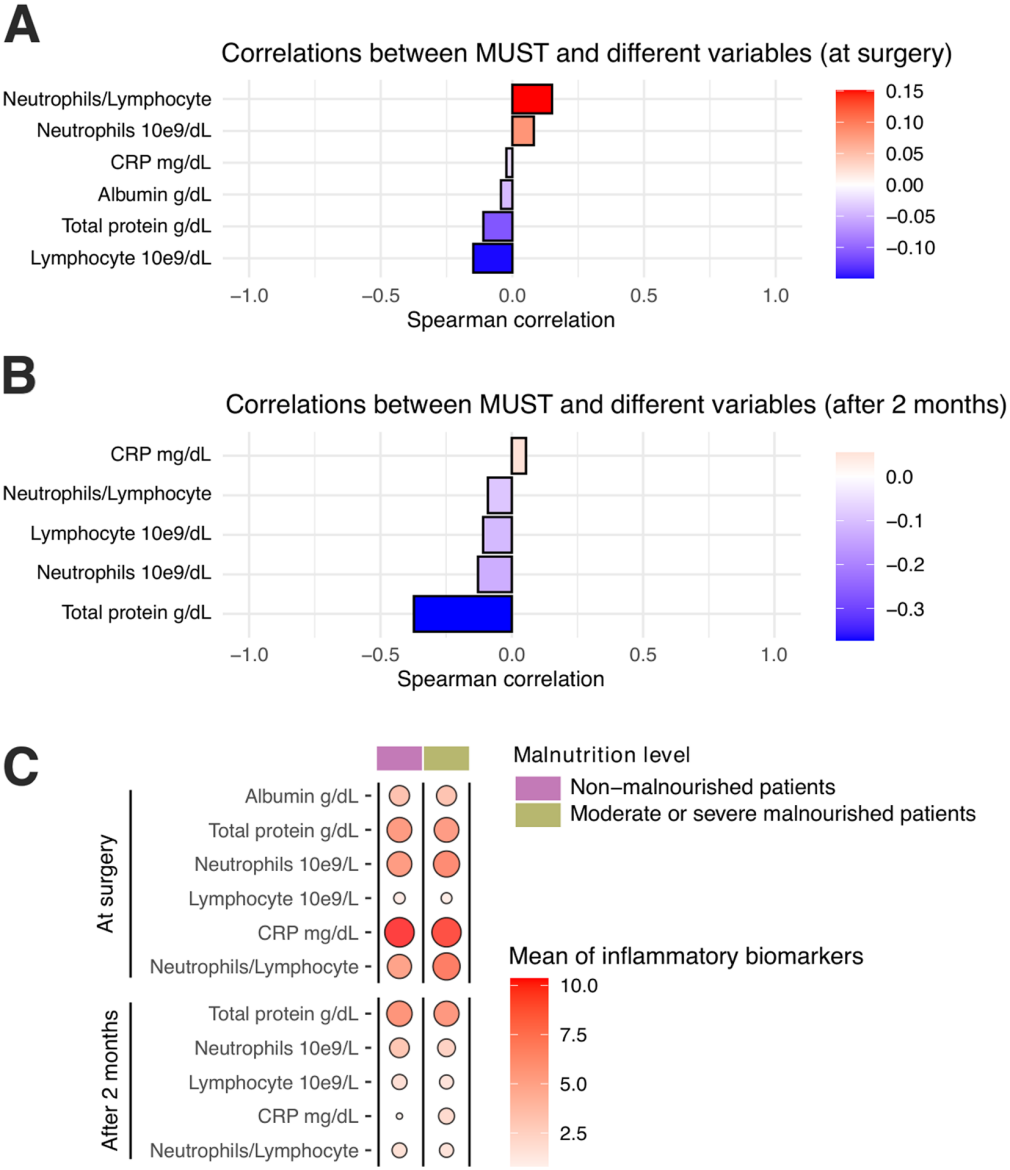
**(A)** Bar plot representing mean frequency and standard error of iNKT cells producing different cytokines in tumor (TUM) samples of non-malnourished (violet) and moderate or severe malnourished patients (green). **(B)** Bar plot representing mean frequency and standard error of iNKT cells expressing different activation/exhaustion molecules in tumor (TUM) samples of non-malnourished (violet) and moderate or severe malnourished patients (green). **(C)** Bar plot representing mean frequency and standard error of iNKT cells producing different cytokines in adjacent non-tumor colon tissue (NCT) of non-malnourished (violet) and moderate or severe malnourished patients (green). **(D)** Bar plot representing mean frequency and standard error of iNKT cells expressing different activation/exhaustion molecules in adjacent non-tumor colon tissue (NCT) of non-malnourished (violet) and moderate or severe malnourished patients (green). Nominal *p*-values from Mann Whitney test are reported only if significant (≤ 0.05).

In cancer, CD4 + T cells are pivotal, with the Th1 subtype contributing to anti-tumor immunity through the secretion of IFNγ and TNF-*α*, and Th2 cells secreting pro-tumoral, anti-inflammatory mediators (39). Their role in immune checkpoint blockade efficacy is also gaining attention (39). Focusing on CD4^+^ T cell functionality, we found that nutritional status did not significantly impact CD4^+^ T cell activity in CRC patients within our cohort. Nonetheless, malnourished patients tended to exhibit a non-significant reduction in secretion of TNFα, IFNγ, and IL-10 (Figure 4A), accompanied by a non-significant increment in IL-17 secretion within TUMs (Figure 4A). In NCTs, only IFNγ levels showed a reduction, even if not significant, in malnourished individuals (Figure 4C). Additionally, CD4^+^ T cells in TUMs from malnourished patients showed a non-significant increase in the expression of exhaustion markers (i.e., CTLA-4, PD-1, and TIGIT), together with a reduction in the activation marker CD69, compared to TUMs from non-malnourished patients (Figure 4B). Conversely, in NCTs, CD4^+^CD69^+^ T cells tended to be lower in non-malnourished patients compared to malnourished individuals, although this difference did not reach statistical significance (Figure 4D). Similar results were observed in patients divided in three groups by the MUST score, where severe malnourished patients showed significant increase of CD4^+^PD1^+^ and CD4^+^CD69^+^ T cells in NCTs (Supplementary Figures 6A–D). Collectively, these findings suggest that malnutrition may be linked to differences in CD4 + T cell functional profiles. Invariant NKT (iNKT) cells, known for bridging innate and adaptive immunity, have diverse roles in cancer, from direct tumor killing to modulating immune responses (39). However, their function in CRC remains unclear (14, 40). Cytokine profiling in this cohort mirrored the patterns observed in the CD4^+^ T cell compartment (Figure 5).

Specifically, malnourished patients showed reduced production of TNFα, IFNγ, IL-13, and IL-10 in both TUM and NCT tissues (Figures 5A,C) especially when patients are divided in three groups (Supplementary Figures 7A–D). Notably, while IL-17 levels decreased in NCTs from malnourished patients, they were elevated in TUMs from malnourished individuals. Additionally, increased levels of IL-4 and GM-CSF were detected in both intestinal regions of malnourished patients, suggesting a shift toward a pro-tumorigenic cytokine milieu (Figure 5C). It is worth noting that only IFNγ and IL-4 alterations in NCT tissues reached statistical significance in malnourished patients. Furthermore, moderate to severe malnutrition tended to show higher expression levels of immune checkpoint molecules (CTLA-4, PD-1, TIGIT, and TIM-3) on iNKT cells (Figures 5B,D), even if not statistically significant. Collectively, these data indicate that iNKT cells from malnourished patients display features consistent with a more pro-tumorigenic immune milieu, including increased IL-17 production, reduced IFNγ levels, enhanced IL-4 secretion, and increased expression of exhaustion markers.

### Nutritional status is associated with microbiota patterns related to immune suppression in the TME

**Figure 6:**
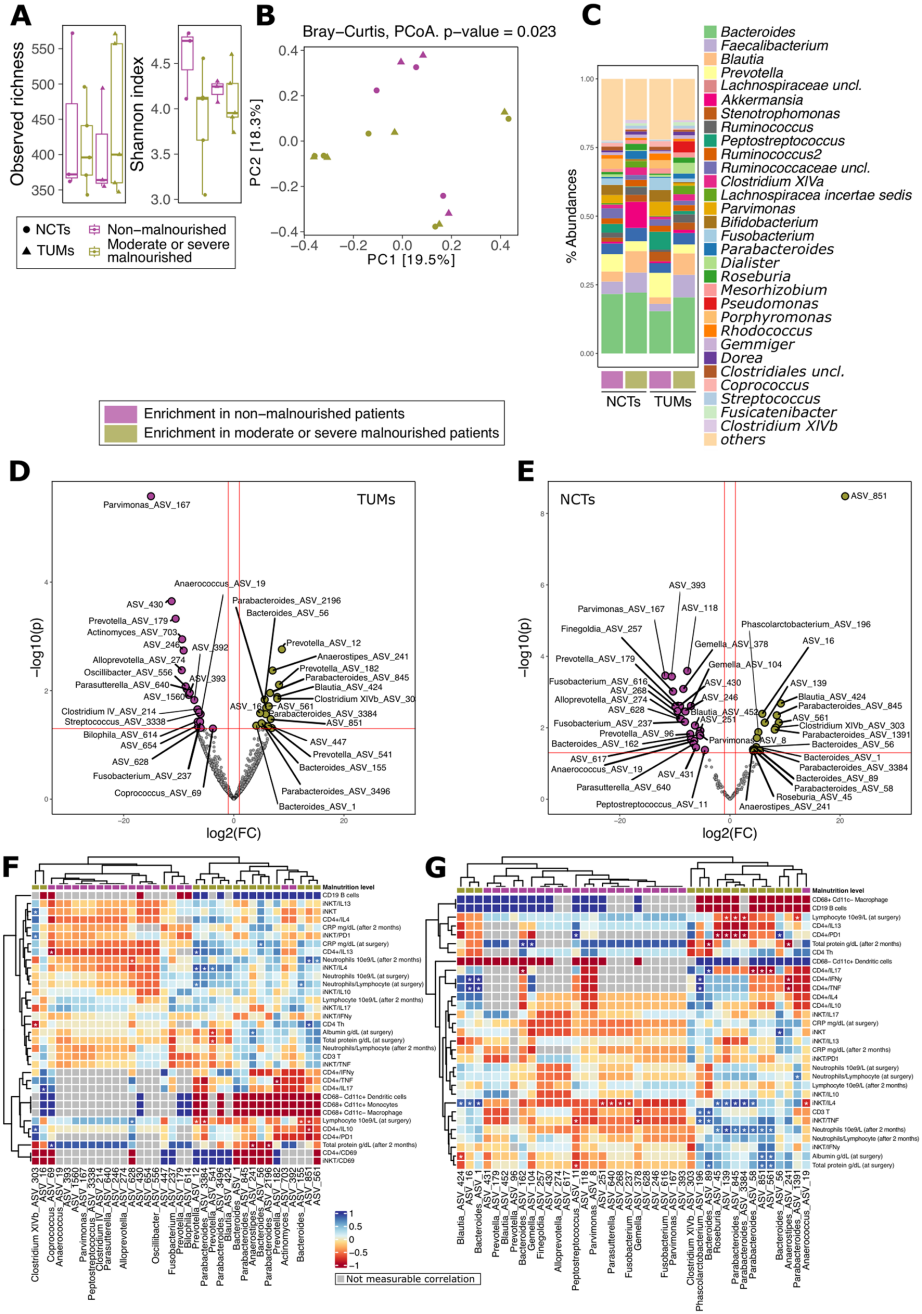
**(A)** Microbiota *α*-diversity as observed richness and as measured by Shannon index in tumoral and distal regions of non-malnourished and moderate/severe malnourished CRC patients. **(B)** PCoA of microbial *β*-diversity as measured by Bray-Curtis (*p*-value from PERMANOVA test). **(C)** Bar plot representing relative abundances of the most abundant bacterial taxa in tumor (TUM) and adjacent non-tumor colon tissue (NCT) samples of patients grouped by level of malnutrition. **(D)** Volcano plot showing the ASVs significantly enriched in non-malnourished or moderate/severe malnourished patients, considering tumor (TUM) samples. **(E)** Volcano plot showing the ASVs significantly enriched in non-malnourished or moderate/severe malnourished patients, considering adjacent non-tumor colon tissue (NCT) samples. **(F)** Heatmap of Spearman’s correlations between the relative abundance of the ASVs enriched in non-malnourished or moderate/severe malnourished patients with the values of biochemical inflammatory markers and the frequency of infiltrating immune cells, considering tumor (TUM) samples. Asterisk for correlations with a nominal *p*-value ≤ 0.05 from *t*-test. **(G)** Heatmap of Spearman’s correlations between the relative abundance of the ASVs enriched in non-malnourished or moderate/severe malnourished patients with the values of biochemical inflammatory markers and the frequency of infiltrating immune cells, considering adjacent non-tumor colon tissue (NCT) samples. Asterisk for correlations with a nominal *p*-value ≤ 0.05 from *t*-test.

The gut microbiome has been indicated as a key driver of the development and progression of CRC (41). To explore its link with nutritional status and clinical outcomes, we analyzed the tumor-associated microbiota. Of the 43 patients included in the study, the mucosal microbiota (i.e., isolated from the surface of the tumor and the corresponding NCT region) was available for a subset of 8 patients, including 5 malnourished and 3 non-malnourished individuals. Overall, 1,515 different ASVs were identified in malnourished patients, while 1,173 in non-malnourished patients; among them, 750 ASVs were in common between the two groups. While the alpha diversity was not significantly different neither depending on the level of malnutrition nor on the type of tissue (Figure 6A), the beta diversity, estimated as Bray-Curtis distance between samples, was significantly different (*p*-value = 0.023 from PERMANOVA test) between malnourished and non-malnourished patients (Figure 6B). Moreover, we observed a specific nutrition-based enrichment of different microorganisms. Malnourished patients showed a higher abundance of *Akkermansia* (mainly in NCTs) and *Blautia*, while non-malnourished patients had greater levels of *Fusobacterium* and *Peptostreptococcus*, especially in TUMs (Figure 6C). Further comparisons of tumor- and NCT-derived microbiota revealed that *Bacteroides*, *Prevotella*, and *Parabacteroides* were enriched in malnourished patients, whereas *Oscillibacter*, *Alloprevotella*, and *Finegoldia* were more prevalent in non-malnourished individuals (Figures 6D,E). Given the known role of the gut microbiota in shaping immune function and cancer progression (39), we conducted correlation analyses to explore links between microbial composition, immune parameters, and nutritional status (Figures 6F,G). Notably, bacteria enriched in malnourished patients, *Bacteroides* and *Prevotella* in tumors (Figure 6F), and *Parabacteroides* in NCTs (Figure 6G), showed a strong positive correlation with the NLR at the time of surgery, pointing to a potential microbiota-driven mechanism promoting systemic inflammation. In contrast, the bacteria enriched in non-malnourished patients, such as *Oscillibacter*, *Alloprevotella*, and *Finegoldia*, displayed a negative, although not statistically significant, correlation with NLR.

These findings align with additional immune correlations: *Prevotella* negatively associated with CD4^+^ TNFα^+^ cells in TUMs (Figure 6F), while *Bacteroides* in NCTs positively correlated with CD4^+^ PD-1^+^ and IL-17^+^ cells in malnourished patients, but negatively with IL-17^+^ cells in non-malnourished individuals (Figure 6G).

We also uncovered associations between microbiota composition and iNKT cell activity. In malnourished patients, IL-4^+^ iNKT cells positively correlated with *Blautia*, *Bacteroides*, *Parabacteroides*, and *Roseburia*. Conversely, in non-malnourished patients, they negatively correlated with *Fusobacterium*, *Parasutterella*, and *Anaerococcus* (Figure 6G).

Taken together, these findings highlight how nutritional status may play a role in specific shifts in the tumor-associated microbiota, which in turn may shape the immune landscape, particularly by enhancing systemic inflammation as reflected by elevated NLR, and potentially impact the clinical trajectory of CRC patients undergoing surgery.

## Discussion

Malnutrition significantly compromises cancer therapy by reducing treatment efficacy, increasing toxicity, impairing immunotherapy outcomes, and diminishing overall quality of life. It also raises postoperative risks and threatens long-term survival (42). Our findings indicate that cancer-related malnutrition is linked to differences in immune function, the TME, and gut microbiota composition in patients with CRC. Although malnutrition is often observed in older (65+) male patients and in association with advanced cancer stages (74.7%), its demographic distribution remains a subject of ongoing debate (43, 44). Interestingly, our study found no significant differences in nutritional status based on sex, age, or disease stage (Figure 1). However, measurable reductions in BMI and body weight were evident among patients identified as moderately or severely malnourished (Supplementary Figure 2), reinforcing the value of routine anthropometric assessment tools, such as the MUST, in CRC patient evaluation. Beyond physical metrics, our data reveal that malnutrition is tightly linked to systemic inflammation. Consistent with prior reports (40), malnourished CRC patients exhibited a marked increase in tissue-infiltrating neutrophils, particularly in the non-tumoral colonic tissue (NCT) (Figure 3B; Supplementary Figure 5B). Neutrophils, once considered mere responders to infection, are now recognized as active participants in tumor progression. They contribute to angiogenesis, matrix remodelling, metastasis, and, crucially, immune suppression. A key mechanism involves the inhibition of T cell activation, which weakens the adaptive immune response and facilitates immune evasion (45).

In line with this, our study suggests a pattern of immune dysfunction within the T cell compartment of malnourished patients. These individuals exhibited diminished, even if not significant, anti-tumor cytokine production (e.g., IFN-*γ*, TNF-*α*) and a skewing toward a pro-tumoral cytokine profile (e.g., IL-4, IL-17). The upregulation in malnourished patients of immune checkpoint molecules such as PD-1, CTLA-4, TIGIT, and TIM-3 on CD4^+^ T cells and iNKT cells further pointed to possible impaired effector function of T cell compartment (Figures 4, 5). Most of these changes were particularly pronounced in the NCT, where neutrophil accumulation in malnourished patients was highest (Figure 3), suggesting a spatial link between neutrophilic inflammation and localized T cell suppression. Given their relative proximity the peritumoral tissue can have an impact on the tumor microenvironment, bearing unique features that could promote tumor progression. Indeed, a high infiltration of neutrophils in the area adjacent to the tumor has been shown to be a negative prognostic factor in hepatocellular carcinoma patients, given the role of these cells in shaping the peri-tumoral and tumoral microenvironment (46).

Importantly, these immune alterations may not be solely attributable to neutrophils. Nutritional status itself is a key modulator of immune competence. In murine models, fasting has been shown to reduce CD4^+^ T cell counts and cytokine production, including IFN-γ and IL-17, indicating impaired Th1 and Th17 responses (43). The metabolic fitness of T cells is tightly linked to their ability to differentiate into functional effector subsets; glucose and glutamine are essential for this process. Disruptions in cellular metabolism, such as impaired glycolysis, hinder T cell differentiation and function (44, 45).

The gut microbiota, an essential interface between diet and immunity, also plays a critical role in this equation. Malnutrition has long been associated with gut microbial dysbiosis (46), and fecal microbiota transplants from malnourished donors have been shown to promote tumor growth in preclinical models (47). The microbiota plays a pivotal role in modulating the immune system, and increasing evidence indicates that bacteria residing in peritumoral regions may also contribute to tumor progression. Notably, *Porphyromonas gingivalis*, although found to be enriched in distal sites of CRC patients, has been demonstrated to exert pro-tumorigenic effects (47). It is well established that the gut microbiota of CRC patients differs from that of healthy individuals, with distinct compositional and functional alterations described across the adenoma-carcinoma sequence (48–50). Therefore, the present study was not designed to compare CRC patients with healthy controls, but rather to investigate whether, within the CRC population, nutritional status may represent an additional stratifying factor associated with distinct immunological and microbiota profiles.

In our study, microbiota composition differed significantly based on patients’ nutritional status. In malnourished individuals, we observed an increased abundance of *Bacteroides*, *Prevotella* in tumor regions, and *Parabacteroides* in adjacent non-tumor tissue (Figures 6C–E), all of which were positively correlated with NLR at surgery (Figure 6F). These bacterial genera have been previously linked to neutrophil recruitment in inflammatory contexts (51–53). Their expansion in malnourished patients may therefore contribute to a self-sustaining cycle of neutrophil-driven inflammation and immune suppression. Mechanistically, species belonging to *Prevotella* and *Bacteroides* have been reported to stimulate dendritic cells via TLR2 signaling and to promote the release of IL-1β, IL-6, and IL-23, thereby enhancing Th17 polarization and IL-17 production. These cytokine pathways are known to support neutrophil recruitment and activation within inflammatory tissues. In this context, the enrichment of these taxa in malnourished patients may represent a microbiota-associated amplification loop potentially contributing to the neutrophil-associated immune signatures observed in our cohort (54, 55).

Conversely, taxa such as *Oscillibacter* and *Alloprevotella*, which showed a non-significant negative correlation with NLR, may represent protective microbiota signatures worth further investigation.

Microbial activity not only impacted neutrophils but also played a significant role in shaping T cell responses. The presence of certain bacterial species, including *Firmicutes*, *Proteobacteria*, and *Bacteroides*, has been associated with the induction of chemokines that recruit T cells to the tumor site (50). In contrast, *Fusobacteria*, despite being linked to poor prognosis, can still stimulate immune cell-attracting chemokines *in vitro* (50). In our study, associations between specific microbes and cytokine-producing CD4^+^ and iNKT cells suggest that the microbiota not only modulates inflammation, but also helps shaping the nature of anti-tumor immunity.

Despite these insights, the specific roles of individual bacterial species in regulating host immunity and nutritional status remain to be clarified. What is evident, however, is the existence of a potent immunometabolic axis linking malnutrition, neutrophil-driven inflammation, and microbial dysbiosis that collectively weakens immune defenses and fuels the progression of CRC.

These findings strongly support the integration of nutritional assessments into standard oncological care. Early detection of malnutrition through validated screening tools can enable timely and targeted interventions, potentially restoring immune competence, improving treatment responses, and ultimately extending patient survival.

In conclusion, our study suggests the existence of a complex and clinically meaningful interplay between malnutrition, immune suppression, and gut microbiota imbalance in CRC. However, due to the relatively small size of the cohort of patients and the intrinsic variability of immune cell infiltrates and cytokine expression, these results should be regarded as exploratory. Therefore, the findings should be interpreted as hypothesis-generating rather than definitive. We observed trends in immune responses and microbiota composition between malnourished and non-malnourished patients. Although many of these differences did not reach statistical significance, they may reflect biological patterns that warrant further evaluation in larger, independent cohorts. Future studies with greater statistical power and standardized analytical approaches will be essential to validate and expand upon these preliminary observations. Indeed, recognizing malnutrition as a modifiable risk factor, and addressing it within a personalized care framework, may open new avenues to boost anti-tumor immunity and enhance therapeutic efficacy in CRC. Nonetheless, further mechanistic studies are essential to unravel how diet, metabolism, and microbial communities converge to shape immune function in the context of cancer.

## Data Availability

The datasets presented in this study can be found in online repositories. The names of the repository/repositories and accession number(s) can be found in the article/Supplementary material.

## Supplementary material

SUPPLEMENTARY FIGURE 1 **(A)** Bar plot representing the relative distribution of male (blue) and female (pink) in non-malnourished patients (*n* = 24), moderate malnourished patients (*n* = 10) and severe malnourished patients (*n* = 9). **(B)** Bar plot representing the numbers of non-malnourished (yellow), moderate malnourished (orange) and severe malnourished patients (red) in the four stages of the disease (I *n* = 3, II *n* = 10, III *n* = 17, IV *n* = 3). **(C)** Violin plot representing the age distribution in non-malnourished patients (*n* = 24), moderate malnourished patients (*n* = 10) and severe malnourished patients (*n* = 9). **(D)** Violin plot representing the age at diagnosis distribution in non-malnourished patients (*n* = 24, yellow), moderate malnourished patients (*n* = 10, orange) and severe malnourished patients (*n* = 9, red).

SUPPLEMENTARY FIGURE 2 **(A)** Violin plot representing the BMI distribution in non-malnourished patients (*n* = 24, violet) and in moderate or severe malnourished patients (*n* = 19, green). **(B)** Violin plot representing the weight distribution in non-malnourished patients (n = 24, violet) and in moderate or severe malnourished patients (*n* = 19, green). **(C)** Violin plot representing the BMI distribution in non-malnourished patients (*n* = 24, yellow), moderate malnourished patients (*n* = 10, orange) and severe malnourished patients (*n* = 9, red). **(D)** Violin plot representing the weight distribution in non-malnourished patients (*n* = 24, yellow), moderate malnourished patients (*n* = 10, orange) and severe malnourished patients (*n* = 9, red).

SUPPLEMENTARY FIGURE 3 Comprehensive representation of the mean values of different biochemical markers of inflammation, measured at the time of surgical intervention and two months after, in patients grouped by level of malnutrition.

SUPPLEMENTARY FIGURE 4 **(A)** Information and statistics regarding the Logistic Regression model trained to predict the level of malnutrition. Different demographical and biochemical parameters were selected using Lasso regression run with 10-folds cross validation. **(B)** The model has sensitivity = 0.74, specificity = 1, misclassification rate = 0.09, accuracy = 0.91, precision = 1, *p* = 0.01, F1 score = 0.85, AIC = 56.6, BIC = 67.27, pseudo-R² (McFadden) = 0.24, pseudo-R² (Cragg-Uhler) = 0.38, *p* = 0.01, χ²(5) = 14.33. The AUC is 0.92.

SUPPLEMENTARY FIGURE 5 **(A)** Bar plot representing mean frequency and standard error of different immune cellular population analyzed by FACS infiltrating the tumor samples (TUM) of non-malnourished (yellow), moderate (orange) and severe malnourished (red) patients. **(B)** Bar plot representing mean frequency and standard error of different immune cellular population analyzed by FACS infiltrating the adjacent non-tumor colon tissue (NCT) of non-malnourished (yellow), moderate (orange) and severe malnourished (red) patients. Nominal p-values from Mann Whitney test are reported only if significant (≤ 0.05).

SUPPLEMENTARY FIGURE 6 **(A)** Bar plot representing mean frequency and standard error of CD4^+^ cells producing different cytokines in tumor samples (TUM) of non-malnourished (yellow), moderate (orange) and severe malnourished (red) patients. **(B)** Bar plot representing mean frequency and standard error of CD4^+^ cells expressing different activation/exhaustion molecules in tumor samples (TUM) of non-malnourished (yellow), moderate (orange) and severe malnourished (red) patients. **(C)** Bar plot representing mean frequency and standard error of CD4^+^ cells producing different cytokines in adjacent non-tumor colon tissue (NCT) of non-malnourished (yellow), moderate (orange) and severe malnourished (red) patients. **(D)** Bar plot representing mean frequency and standard error of CD4^+^ cells expressing different activation/exhaustion molecules in adjacent non-tumor colon tissue (NCT) of non-malnourished (yellow), moderate (orange) and severe malnourished (red) patients. Nominal p-values from Mann Whitney test are reported only if significant (≤ 0.05).

SUPPLEMENTARY FIGURE 7 **(A)** Bar plot representing mean frequency and standard error of iNKT cells producing different cytokines in tumor samples (TUM) of non-malnourished (yellow), moderate (orange) and severe malnourished (red) patients. **(B)** Bar plot representing mean frequency and standard error of iNKT cells expressing different activation/exhaustion molecules in tumor samples (TUM) of non-malnourished (yellow), moderate (orange) and severe malnourished (red) patients. **(C)** Bar plot representing mean frequency and standard error of iNKT cells producing different cytokines in adjacent non-tumor colon tissue (NCT) of non-malnourished (yellow), moderate (orange) and severe malnourished (red) patients. **(D)** Bar plot representing mean frequency and standard error of iNKT cells expressing different activation/exhaustion molecules in adjacent non-tumor colon tissue (NCT) of non-malnourished (yellow), moderate (orange) and severe malnourished (red) patients. Nominal p-values from Mann Whitney test are reported only if significant (≤ 0.05).

SUPPLEMENTARY FIGURE 8 Flow diagram of patient screening, inclusion, and analytical subsets. Among 118 patients undergoing colon cancer resection, 43 were included in nutritional assessment (MUST), biochemical evaluation, and immune phenotyping (FACS analysis of tumor [TUM] and adjacent non-tumor colon tissue [NCT]). Microbiota profiling (16S rRNA sequencing) was performed in a subset of 8 patients for whom mucosal samples were available (5 malnourished and 3 non-malnourished).
