# Supplementary figures and images for: Preoperative malnutrition is associated with suppressed intratumoral T cell function and distinct tumor-associated microbiota in colorectal cancer: a prospective pilot study
Source: Front Nutr. 2026 May 28;13:1802354. doi: 10.3389/fnut.2026.1802354 (PMC13274497; doi:10.3389/fnut.2026.1802354)

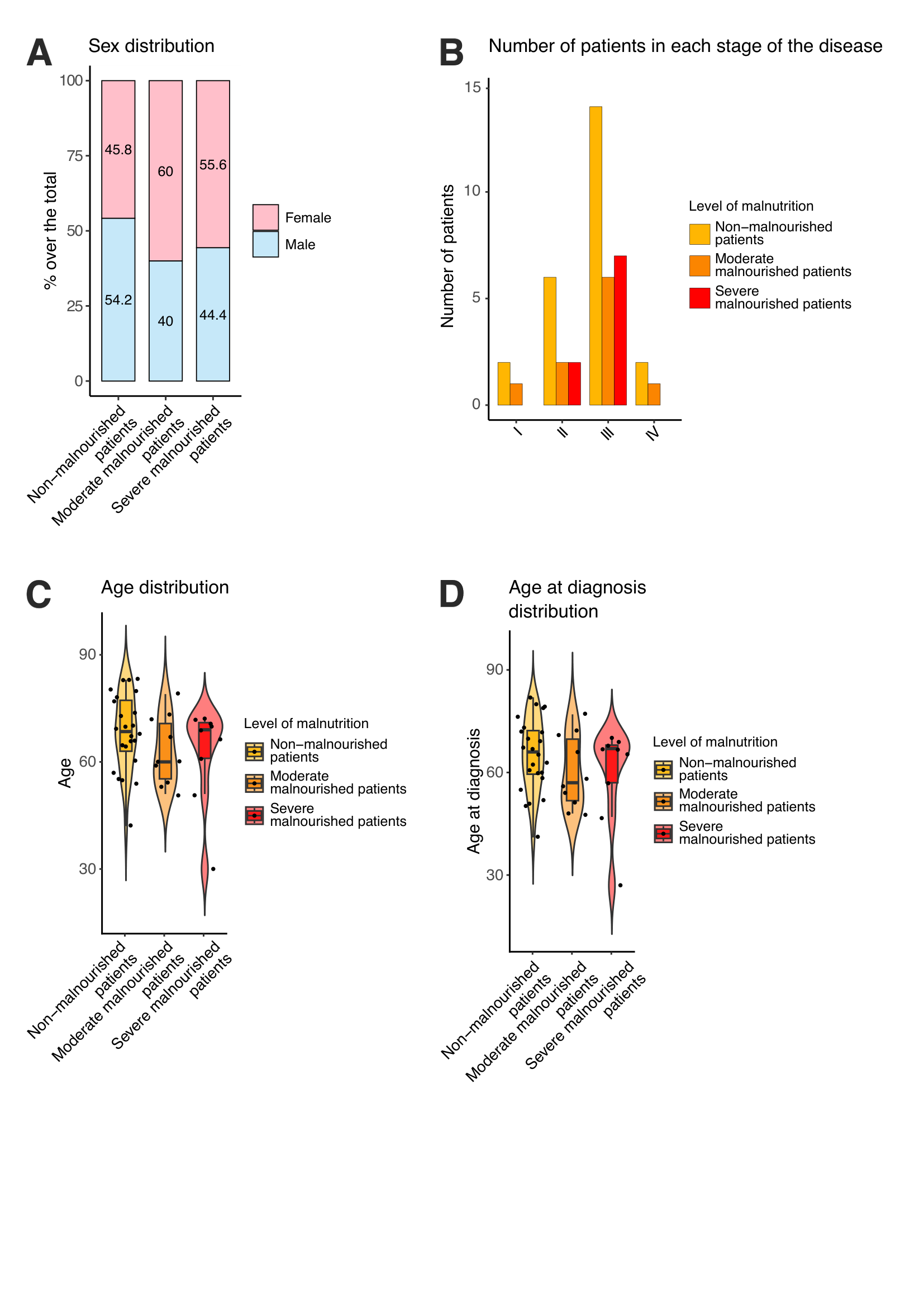

Supplement: SUPPLEMENTARY FIGURE 1 — (A) Bar plot representing the relative distribution of male (blue) and female (pink) in non-malnourished patients (n = 24), moderate malnourished patients (n = 10) and severe malnourished patients (n = 9). (B) Bar plot representing the numbers of non-malnourished (yellow), moderate malnourished (orange) and severe malnourished patients (red) in the four stages of the disease (I n = 3, II n = 10, III n = 17, IV n = 3). (C) Violin plot representing the age distribution in non-malnourished patients (n = 24), moderate malnourished patients (n = 10) and severe malnourished patients (n = 9). (D) Violin plot representing the age at diagnosis distribution in non-malnourished patients (n = 24, yellow), moderate malnourished patients (n = 10, orange) and severe malnourished patients (n = 9, red). [file Image_1.tiff]

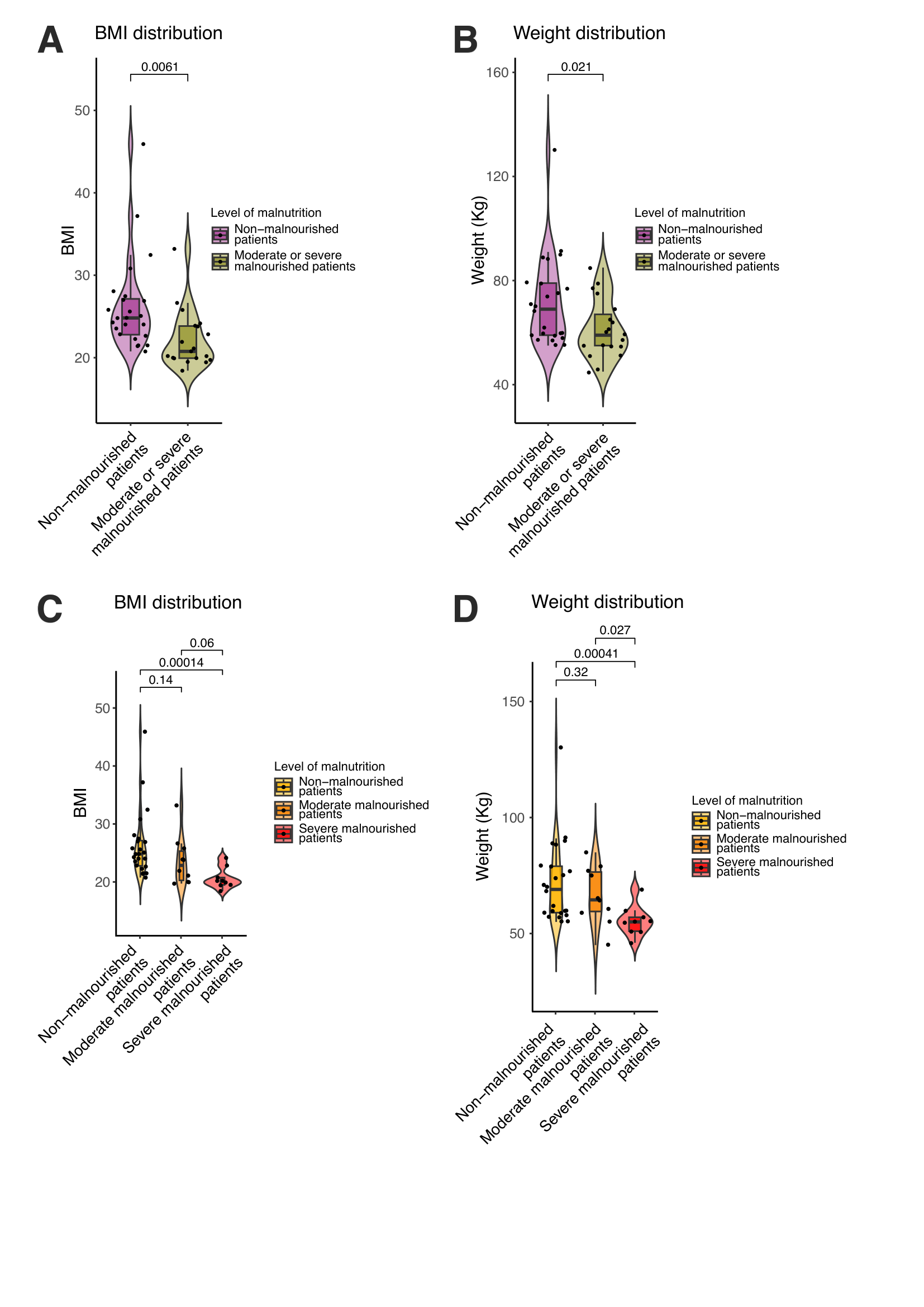

Supplement: SUPPLEMENTARY FIGURE 2 — (A) Violin plot representing the BMI distribution in non-malnourished patients (n = 24, violet) and in moderate or severe malnourished patients (n = 19, green). (B) Violin plot representing the weight distribution in non-malnourished patients (n = 24, violet) and in moderate or severe malnourished patients (n = 19, green). (C) Violin plot representing the BMI distribution in non-malnourished patients (n = 24, yellow), moderate malnourished patients (n = 10, orange) and severe malnourished patients (n = 9, red). (D) Violin plot representing the weight distribution in non-malnourished patients (n = 24, yellow), moderate malnourished patients (n = 10, orange) and severe malnourished patients (n = 9, red). [file Image_2.tiff]

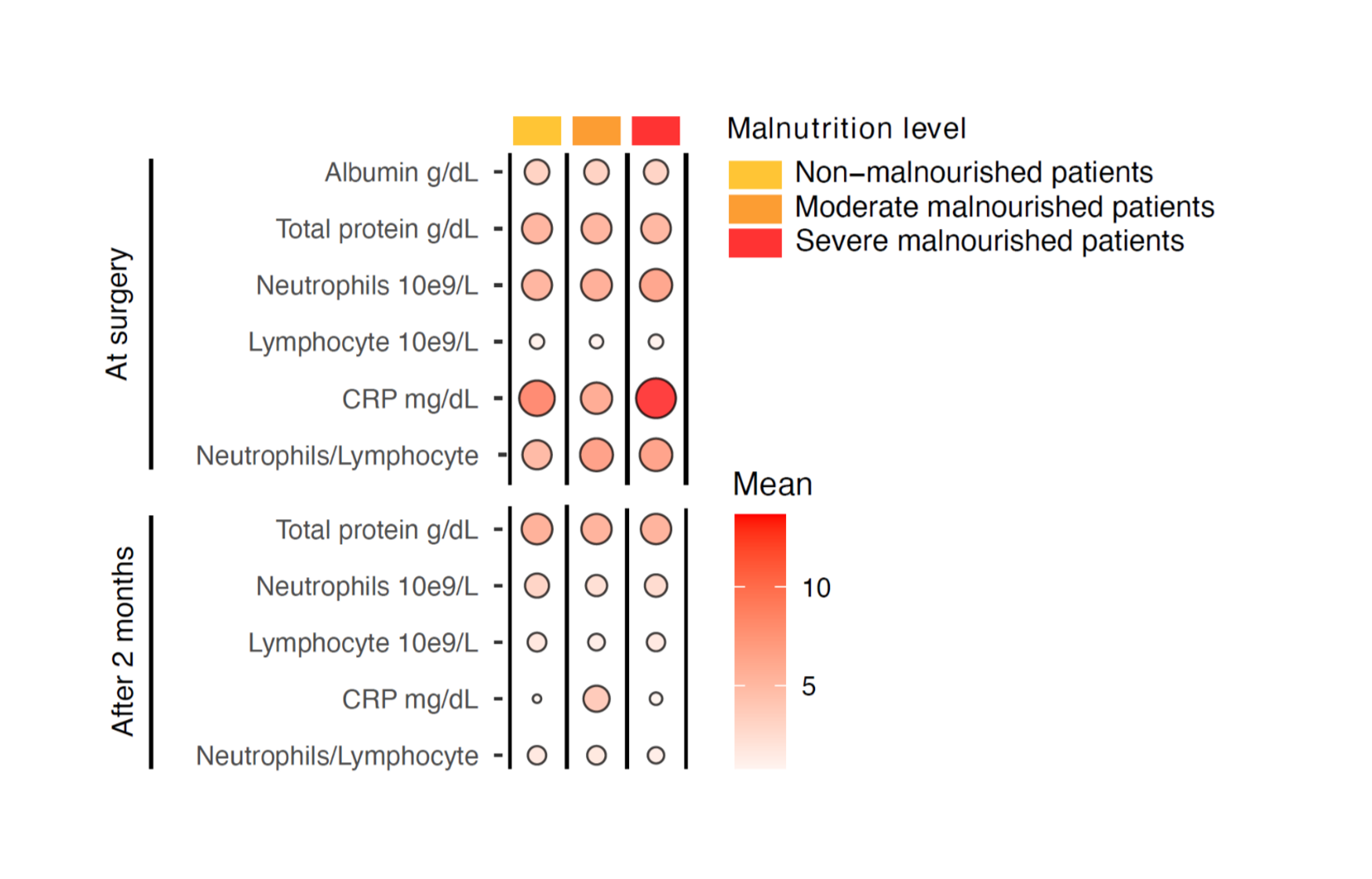

Supplement: SUPPLEMENTARY FIGURE 3 — Comprehensive representation of the mean values of different biochemical markers of inflammation, measured at the time of surgical intervention and two months after, in patients grouped by level of malnutrition. [file Image_3.tif]

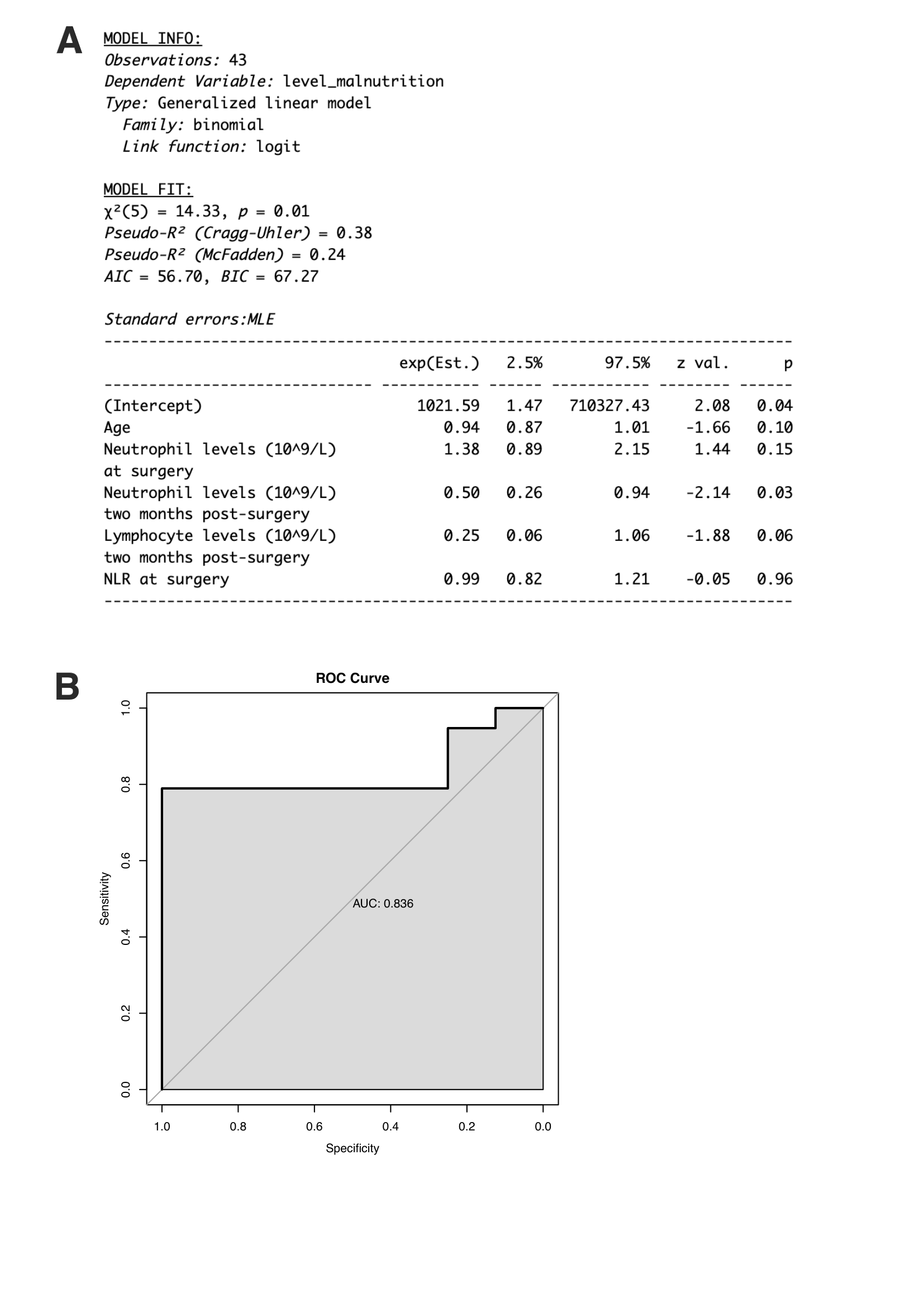

Supplement: SUPPLEMENTARY FIGURE 4 — (A) Information and statistics regarding the Logistic Regression model trained to predict the level of malnutrition. Different demographical and biochemical parameters were selected using Lasso regression run with 10-folds cross validation. (B) The model has sensitivity = 0.74, specificity = 1, misclassification rate = 0.09, accuracy = 0.91, precision = 1, p = 0.01, F1 score = 0.85, AIC = 56.6, BIC = 67.27, pseudo-R² (McFadden) = 0.24, pseudo-R² (Cragg-Uhler) = 0.38, p = 0.01, χ²(5) = 14.33. The AUC is 0.92. [file Image_4.tiff]

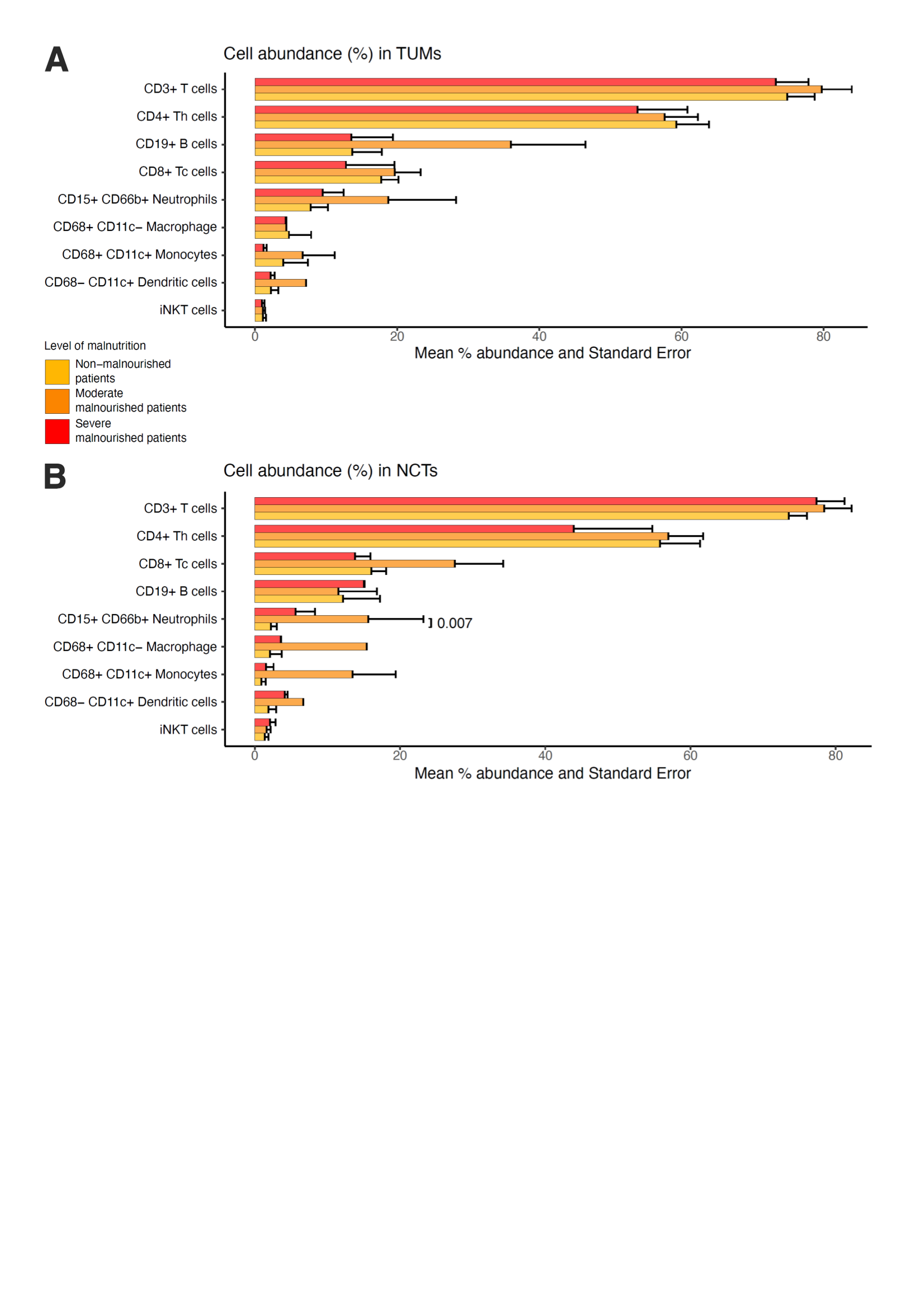

Supplement: SUPPLEMENTARY FIGURE 5 — (A) Bar plot representing mean frequency and standard error of different immune cellular population analyzed by FACS infiltrating the tumor samples (TUM) of non-malnourished (yellow), moderate (orange) and severe malnourished (red) patients. (B) Bar plot representing mean frequency and standard error of different immune cellular population analyzed by FACS infiltrating the adjacent non-tumor colon tissue (NCT) of non-malnourished (yellow), moderate (orange) and severe malnourished (red) patients. Nominal p-values from Mann Whitney test are reported only if significant (≤ 0.05). [file Image_5.tiff]

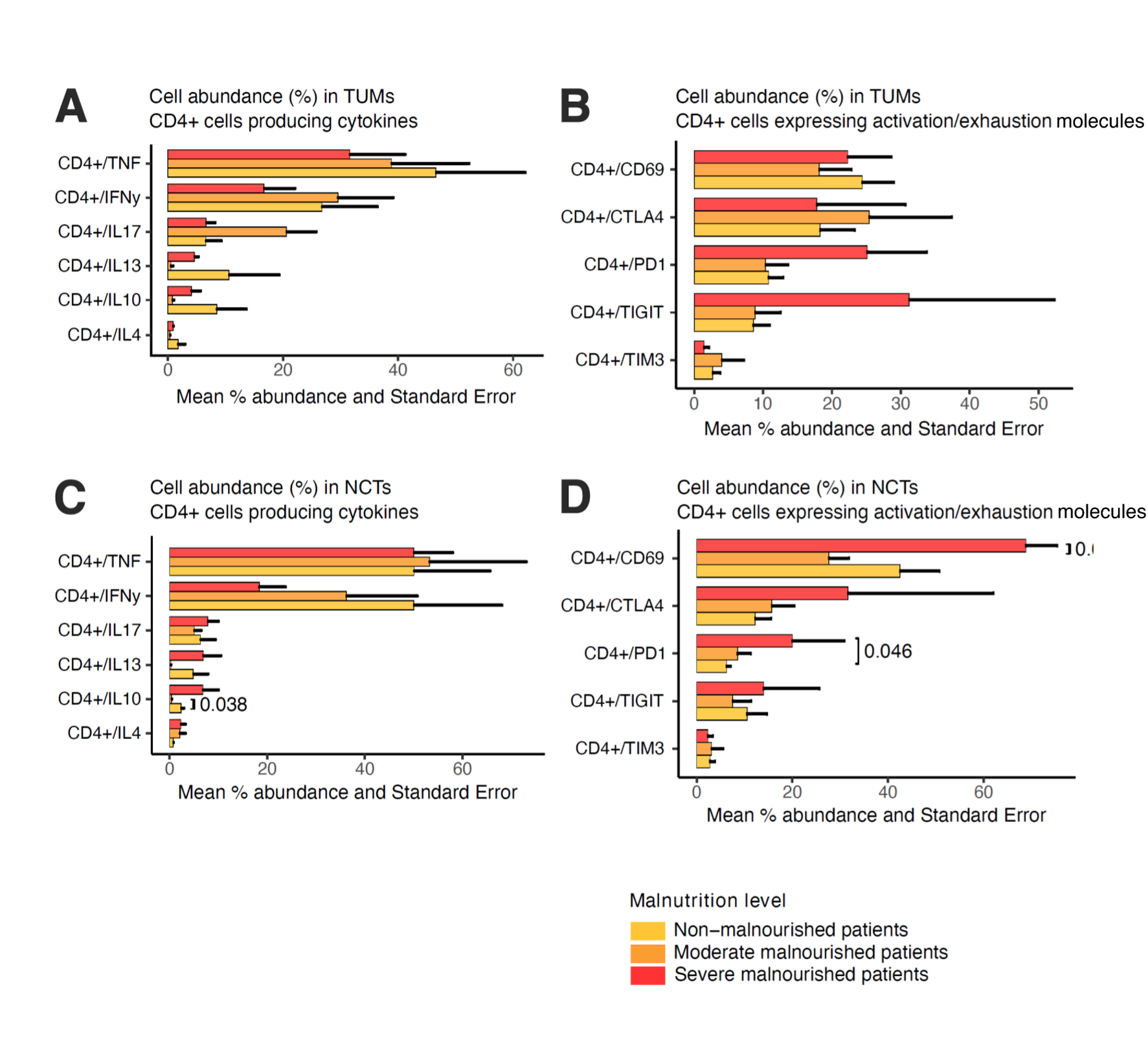

Supplement: SUPPLEMENTARY FIGURE 6 — (A) Bar plot representing mean frequency and standard error of CD4+ cells producing different cytokines in tumor samples (TUM) of non-malnourished (yellow), moderate (orange) and severe malnourished (red) patients. (B) Bar plot representing mean frequency and standard error of CD4+ cells expressing different activation/exhaustion molecules in tumor samples (TUM) of non-malnourished (yellow), moderate (orange) and severe malnourished (red) patients. (C) Bar plot representing mean frequency and standard error of CD4+ cells producing different cytokines in adjacent non-tumor colon tissue (NCT) of non-malnourished (yellow), moderate (orange) and severe malnourished (red) patients. (D) Bar plot representing mean frequency and standard error of CD4+ cells expressing different activation/exhaustion molecules in adjacent non-tumor colon tissue (NCT) of non-malnourished (yellow), moderate (orange) and severe malnourished (red) patients. Nominal p-values from Mann Whitney test are reported only if significant (≤ 0.05). [file Image_6.tif]

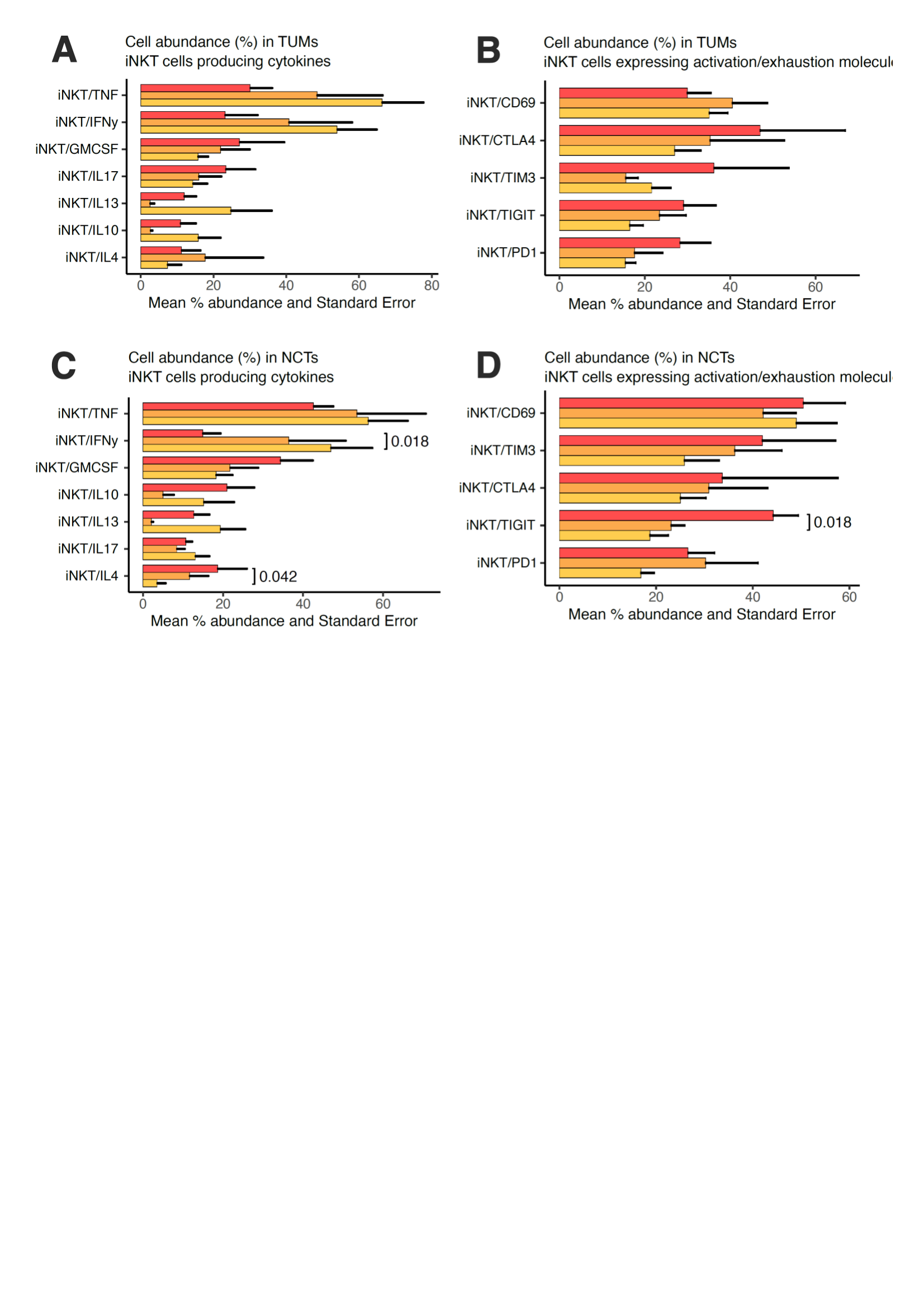

Supplement: SUPPLEMENTARY FIGURE 7 — (A) Bar plot representing mean frequency and standard error of iNKT cells producing different cytokines in tumor samples (TUM) of non-malnourished (yellow), moderate (orange) and severe malnourished (red) patients. (B) Bar plot representing mean frequency and standard error of iNKT cells expressing different activation/exhaustion molecules in tumor samples (TUM) of non-malnourished (yellow), moderate (orange) and severe malnourished (red) patients. (C) Bar plot representing mean frequency and standard error of iNKT cells producing different cytokines in adjacent non-tumor colon tissue (NCT) of non-malnourished (yellow), moderate (orange) and severe malnourished (red) patients. (D) Bar plot representing mean frequency and standard error of iNKT cells expressing different activation/exhaustion molecules in adjacent non-tumor colon tissue (NCT) of non-malnourished (yellow), moderate (orange) and severe malnourished (red) patients. Nominal p-values from Mann Whitney test are reported only if significant (≤ 0.05). [file Image_7.tiff]

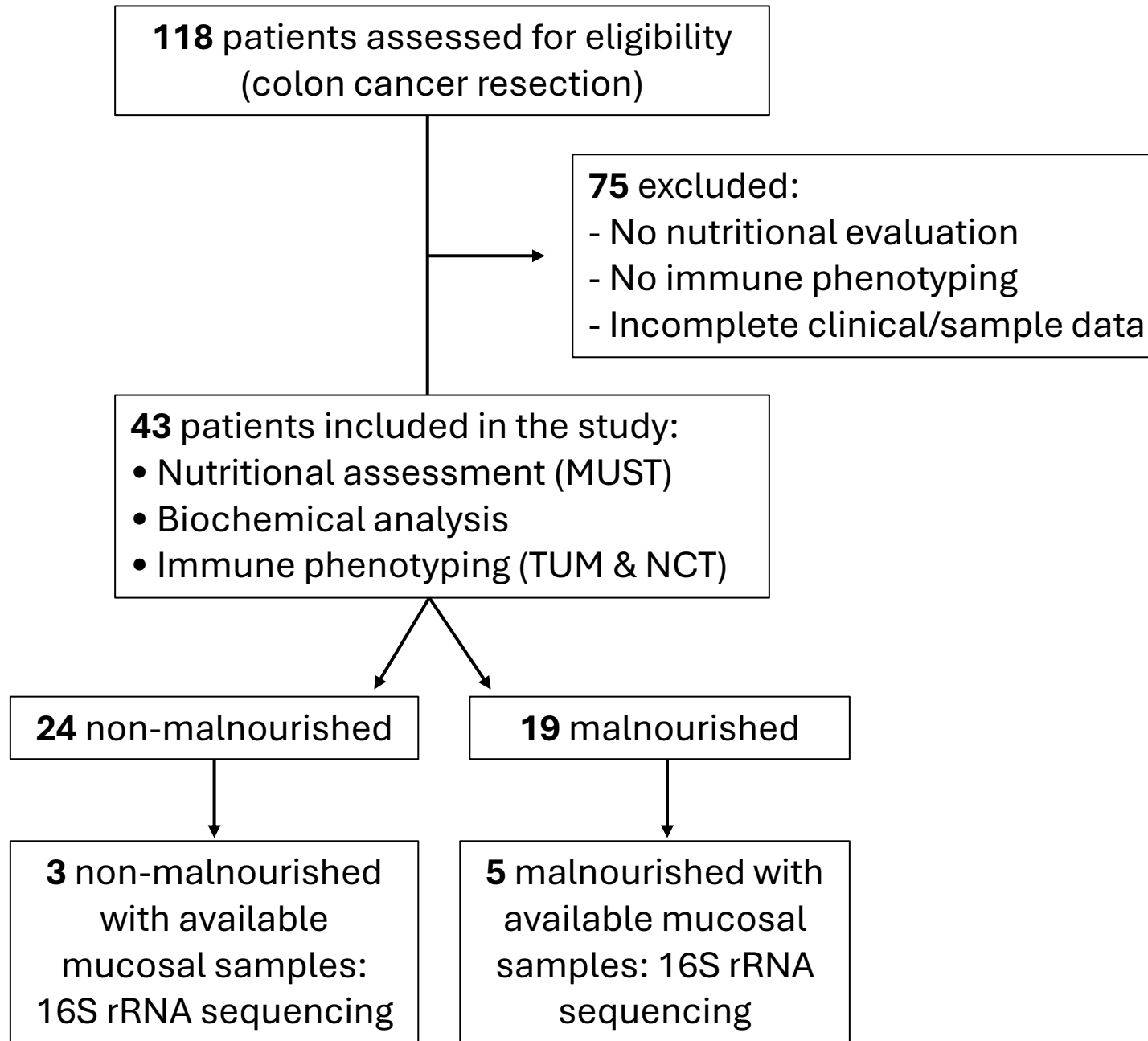

Supplement: SUPPLEMENTARY FIGURE 8 — Flow diagram of patient screening, inclusion, and analytical subsets. Among 118 patients undergoing colon cancer resection, 43 were included in nutritional assessment (MUST), biochemical evaluation, and immune phenotyping (FACS analysis of tumor [TUM] and adjacent non-tumor colon tissue [NCT]). Microbiota profiling (16S rRNA sequencing) was performed in a subset of 8 patients for whom mucosal samples were available (5 malnourished and 3 non-malnourished). [file Image_8.pdf]
