# Supplementary material for: Preoperative malnutrition is associated with suppressed intratumoral T cell function and distinct tumor-associated microbiota in colorectal cancer: a prospective pilot study
Source: Front Nutr. 2026 May 28;13:1802354. doi: 10.3389/fnut.2026.1802354 (PMC13274497; doi:10.3389/fnut.2026.1802354)
Supplement: Supplementary file 9 [file Table_1.docx]

**Table S1: Baseline demographic and clinical characteristics**

| **Variable** | **Total**  **(n=43)** | **Non-malnourished patients**  **(n=24)** | **Moderate malnourished patients**  **(n=10)** | **Severe malnourished patients**  **(n=9)** | **p-value** |
| --- | --- | --- | --- | --- | --- |
|  |  |  |  |  |  |
| Age | 67  (59.50; 72.50) | 68.5  (63; 77.2) | 60  (55.2;70.8) | 69  (61;71) | 0.2982 |
| Age at diagnosis  ≥ 50  < 50 | 65  (55.50;70.50)  38 (88.4%)  5 (11.6%) | 66  (59.5;72.2)  23 (95.8%)  1 (4.2%) | 57  (51.8;69.8)  8 (80%)  2 (20%) | 67  (57;68)  7 (77.8%)  2 (22.2%) | 0.371  0.1672 |
| Sex  Female  Male | 22 (51.2%)  21 (48.8%) | 11 (45.8%)  13 (54.2%) | 6 (60%)  4 (40%) | 5 (55.6%)  4 (44.4%) | 0.7208 |
| BMI | 23.8 (20.93;25.8) | 24.8  (22.8;27.1)  Min 20.8  Max 45.9 | 22.8  (20.3;25.3)  Min 19.7  Max 33.2 | 20.2  (19.5; 20.8)  Min 18.4  Max 24.2 | 0.00074 |
| Weight (Kg) | 61  (57;76) | 69  (59;79)  Min 55  Max 130 | 64.5  (59.5;76.5)  Min 45  Max 85 | 55  (51;57)  Min 46  Max 69 | 0.00472 |
| Tumor localization  Right  Transversal  Left  Rectum | 14 (32.6%)  0  11 (25.6%)  18 (41.8%) | 8 (33.3%)  0  7 (29.2%)  9 (37.5%) | 4 (40%)  0  2 (20%)  4 (40%) | 2 (22.2%)  0  2 (22.2%)  5 (55.6%) | 0.879 |

**Supplementary Table 1**

For continuous variables, the median, first and third quartiles are reported; for categorical variables,

the percentage with respect to the total per group. To test the difference between Non-malnourished patients (MUST = 0), Moderate malnourished patients (MUST = 1,2) and Severe malnourished patients (MUST = 3), the nominal p-value by Kruskal-Wallis was computed for continuous variables, the nominal p-value by Pearson's Chi-squared test with Yates' continuity correction or by Fisher’s exact test was computed for categorical variables.
