# Supplementary material for: Preoperative malnutrition is associated with suppressed intratumoral T cell function and distinct tumor-associated microbiota in colorectal cancer: a prospective pilot study
Source: Front Nutr. 2026 May 28;13:1802354. doi: 10.3389/fnut.2026.1802354 (PMC13274497; doi:10.3389/fnut.2026.1802354)
Supplement: Supplementary file 10 [file Table_2.docx]

**Table S2: Biochemical and immunological**

| **Variable** | **Total**  **(n=43)** | **Non-malnourished patients**  **(n=24)** | **Moderate malnourished patients**  **(n=10)** | **Severe malnourished patients**  **(n=9)** | **p-value** |
| --- | --- | --- | --- | --- | --- |
|  |  |  |  |  |  |
| Albumin g/dL (operation) | 4.34 (3.95;4.53) | 4.2  (4.1;4.56) | 4.4  (3.83;4.5) | 4.27  (3.7;4.53) | 0.905 |
| Total protein g/dL (operation) | 6.9  (6.6;7.15) | 6.9  (6.6;7.01) | 6.85  (6.45;7.18) | 6.75  (6.25,6.95) | 0.608 |
| Total protein g/dL (after 2 months) | 7.35  (6.8;7.58) | 7.55  (6.98;7.68) | 7  (6.8;7.2) | 7.05  (6.93;7.18) | 0.499 |
| Neutrophils 10e9/L (operation) | 6.87  (4.89;8.73) | 6.61  (4.85;8.5) | 6.5  (5.01;8.73) | 7.75  (5.59;8.86) | 0.852 |
| Neutrophils 10e9/L (after 2 months) | 3.15  (2.33;4.26) | 3.22  (2.36;5.43) | 2.76  (2.11;3.93) | 2.61  (2.2;4.17) | 0.463 |
| Lymphocyte 10e9/L (operation) | 1.12  (0.95;1.56) | 1.26  (0.98;1.61) | 0.98  (0.95;1.19) | 1.07  (0.90;1.51) | 0.469 |
| Lymphocyte 10e9/L (after 2 months) | 1.97  (1.54;2.3) | 2.02  (1.6;2.69) | 1.49  (1.15;2.13) | 2.03  (1.63;2.12) | 0.202 |
| CRP mg/dL (operation) | 8.81  (5.94;14.76) | 9.05  (6.32;14.81) | 6.53  (3.6;9.88) | 11.5  (5.17;21.15) | 0.397 |
| CRP mg/dL (after 2 months) | 0.41  (0.13;1.19) | 0.58  (0.11;1.19) | 0.32  (0.18;7.31) | 0.29  (0.18;0.71) | 0.986 |
| Neutrophils/Lymphocyte (operation) | 6.41  (4.03;8.73) | 6.09  (3.75;8) | 7.12  (4.94;12.03) | 6.08  (4.89;7.51) | 0.493 |
| Neutrophils/Lymphocyte (after 2 months) | 1.58  (1.23;2.72) | 1.74  (1.16;2.79) | 1.55  (1.37;3.15) | 1.39  (1.23;1.98) | 0.676 |
| Vascular invasion  Yes  No | 16 (30.77%)  27 (69.23%) | 8 (33.33%)  16 (66.67%) | 4 (40%)  6 (60%) | 4 (44.4%)  5 (55.6%) | 0.8231 |
| Lymphovascular invasion  Yes  No | 1 (2.33%)  42 (97.67) | 0  24 (100%) | 0  10 (100%) | 1 (11.1%)  8 (88.9%) | 0.2093 |
| Perineural invasion  Yes  No | 13 (30.23%)  30 (69.77%) | 6 (25%)  18 (75%) | 4 (40%)  6 (60%) | 3 (33.3%)  6 (66.6%) | 0.6702 |
| Lymphocytic infiltration  Yes  No | 29 (67.44%)  14 (32.56%) | 18 (75%)  6 (25%) | 5 (50%)  5 (50%) | 6 (66.6%)  3 (33.3%) | 0.3755 |

**Supplementary Table 2**

For continuous variables, the median, first and third quartiles are reported; for categorical variables, the percentage with respect to the total per group. To test the difference between Non-malnourished patients (MUST = 0) and Moderate or severe malnourished patients (MUST = 1/2/3), the nominal p-value by Kruskal-Wallis was computed for continuous variables, the nominal p-value by Pearson's Chi-squared test with Yates' continuity correction or by Fisher’s exact test was computed for categorical variables.
