# Supplementary material for: Preoperative malnutrition is associated with suppressed intratumoral T cell function and distinct tumor-associated microbiota in colorectal cancer: a prospective pilot study
Source: Front Nutr. 2026 May 28;13:1802354. doi: 10.3389/fnut.2026.1802354 (PMC13274497; doi:10.3389/fnut.2026.1802354)
Supplement: Supplementary file 11 [file Table_3.docx]

**Table S3. List of FACS Antibodies**

| Name | Cat n. | Source |
| --- | --- | --- |
| Anti-human 279 (PD1) BV650 | Cat.#:564104;Clone:EH12;RRID:AB_2738595 | BD Biosciences |
| Anti-human CD3 APC-Cy7 | Cat.#:25-0038-T100;Clone:UCHT1 | TONBO |
| Anti-human CD3 PE-Cy7 | Cat.#:60-0038-T100;Clone:UCHT1 | TONBO |
| Anti-human CD4 APC-Cy7 | Cat.#:25-0049-T100;Clone:RP A-T4 | TONBO |
| Anti-human CD4 BV605 | Cat.#:300556;Clone:RP A-T4;RRID:AB_2564391 | Biolegend |
| Anti-human CD4 PerCP-Cy5.5 | Cat. #: 317428; Clone: OKT4;RRID: AB_1186122 | Biolegend |
| Anti-human CD45 FITC | Cat. #: 560976; Clone: HI30;RRID: AB_395874 | BD Biosciences |
| Anti-human CD45 BV510 | Cat.#:563204;Clone:HI30;RRID:AB_2738067 | BD Biosciences |
| Anti-human CD69 Alexa Fluor-700 | Cat.#:560739;Clone:FN50;RRID:AB_1727505 | BD Biosciences |
| Anti-human IFN-γ FITC | Cat.#:554551;Clone:4S.B3;RRID:AB_395473 | BD Biosciences |
| Anti-human IL10 PE | Cat.#:501404;Clone JES3-9D7;RRID:AB_315170 | Biolegend |
| Anti-human IL10 BV421 | Cat.#:50142; Clone JES3-9D7;RIDD:AB_2632952 | Biolegend |
| ANTI-HUMAN IL13 PE-CY7 | Cat.#:501914;Clone:JES10-5A2;RRID:AB_2616746 | Biolegend |
| ANTI-HUMAN IL17A APC | Cat.#:11-7179-42;Clone:eBio64DEC17; RRID:AB_1582221 | ThermoFisher Scientific |
| ANTI-HUMAN IL17A FITC | Cat.#:11-717942;Clone:eBio64DEC17; RRID:AB_10805390 | ThermoFisher Scientific |
| ANTI-HUMAN IL22 PERCP-CY5.5 | Cat.#:366710;Clone:2G12A41;RRID:AB_2566794 | Biolegend |
| Anti-human IL4 BV510 | Cat.#:500836;Clone:MP4-25D2;RRID:AB_2650993 | Biolegend |
| ANTI-HUMAN TNF-Α BV785 | Cat.#:502948;Clone:MAb11;RRID:AB_2565858 | Biolegend |
| ANTI-HUMAN GM-CSF PerCPCy5.5 | Cat.#:502312;Clone: BVD2-21C11; RRID:AB_11147946 | Biolegend |
| HUMAN CD1d:BV421 CONJUGATED | N/A | Gift from NIH Tet Facility |
| HUMAN CD1D:PBS-57 TET-BV421 CONJUGATED | N/A | Gift from NIH Tet Facility |
| HUMAN CD1D:PBS-57 TET-PE CONJUGATED | N/A | Gift from NIH Tet Facility |
| HUMAN CD1D:PE CONJUGATED | N/A | Gift from NIH Tet Facility |
| ANTI-HUMAN CD11c BV605 | Cat. #: 301636; Clone: 3.9; RRID:AB_2563796 | Biolegend |
| ANTI-HUMAN CD15 BV785 | Cat. #: 323044; Clone: W6D3; RRID: AB_2632921 | Biolegend |
| ANTI-HUMAN CD152 (CTLA-4) APC | Cat. #: 560938; Clone: BNI3; RRID: AB_398615 | BD Biosciences |
| ANTI-HUMAN CD366 (TIM-3) BV785 | Cat.#:345031;Clone: F38-2E2;RRID: AB_2565833 | Biolegend |
| ANTI-HUMAN CD366 (TIM-3) FITC | Cat.#:345021;;Clone:F38-2E2;RRID: AB_2563936 | Biolegend |
| ANTI-HUMAN CD19 APC | Cat. #: 20-0198-T100;Clone: SJ25C1 | TONBO |
| ANTI-HUMAN CD19 BV650 | Cat. #: 302238; Clone: HIB19;RRID: AB_2562097 | Biolegend |
| ANTI-HUMAN CD66b PerCPCy5.5 | Cat. #: 562254; Clone: G10F5;RRID: AB_11154419 | BD Biosciences |
| ANTI-HUMAN CD68 APC-Cy7 | Cat. #: 333822; Clone: Y1/82A;RRID: AB_2571965 | Biolegend |
| ANTI-HUMAN TIGIT eFluor 450 | Cat. #: 48-9500-42; Clone: MBSA43; RRID: AB_2637414 | ThermoFisher Scientific |
